# Regulating Chondro‐Bone Metabolism for Treatment of Osteoarthritis via High‐Permeability Micro/Nano Hydrogel Microspheres

**DOI:** 10.1002/advs.202305023

**Published:** 2023-12-11

**Authors:** Guilai Zuo, Pengzhen Zhuang, Xinghai Yang, Qi Jia, Zhengwei Cai, Jin Qi, Lianfu Deng, Zhenhua Zhou, Wenguo Cui, Jianru Xiao

**Affiliations:** ^1^ School of Health Science and Engineering University of Shanghai for Science and Technology Shanghai 200093 P. R. China; ^2^ Department of Orthopaedic Oncology Changzheng Hospital Naval Military Medical University Shanghai 200003 P. R. China; ^3^ Department of Bone Tumor The Affiliated Hospital of Qingdao University No. 59, Haier Road Qingdao Shandong 266000 P. R. China; ^4^ Department of Orthopaedics Shanghai Key Laboratory for Prevention and Treatment of Bone and Joint Diseases Shanghai Institute of Traumatology and Orthopaedics Ruijin Hospital Shanghai Jiao Tong University School of Medicine 197 Ruijin 2nd Road Shanghai 200025 P. R. China; ^5^ Pharmaceutical Sciences Laboratory Faculty of Science and Engineering Åbo Akademi University Turku 20520 Finland

**Keywords:** high‐permeability, hydrogel microsphere, osteoarthritis, subchondral bone

## Abstract

Destruction of cartilage due to the abnormal remodeling of subchondral bone (SB) leads to osteoarthritis (OA), and restoring chondro‐bone metabolic homeostasis is the key to the treatment of OA. However, traditional intra‐articular injections for the treatment of OA cannot directly break through the cartilage barrier to reach SB. In this study, the hydrothermal method is used to synthesize ultra‐small size (≈5 nm) selenium‐doped carbon quantum dots (Se‐CQDs, SC), which conjugated with triphenylphosphine (TPP) to create TPP‐Se‐CQDs (SCT). Further, SCT is dynamically complexed with hyaluronic acid modified with aldehyde and methacrylic anhydride (AHAMA) to construct highly permeable micro/nano hydrogel microspheres (SCT@AHAMA) for restoring chondro‐bone metabolic homeostasis. In vitro experiments confirmed that the selenium atoms scavenged reactive oxygen species (ROS) from the mitochondria of mononuclear macrophages, inhibited osteoclast differentiation and function, and suppressed early chondrocyte apoptosis to maintain a balance between cartilage matrix synthesis and catabolism. In vivo experiments further demonstrated that the delivery system inhibited osteoclastogenesis and H‐vessel invasion, thereby regulating the initiation and process of abnormal bone remodeling and inhibiting cartilage degeneration in SB. In conclusion, the micro/nano hydrogel microspheres based on ultra‐small quantum dots facilitate the efficient penetration of articular SB and regulate chondro‐bone metabolism for OA treatment.

## Graphical abstract

Restoring chondro‐bone balance is crucial for OA treatment. Traditional intra‐articular injections cannot directly break through the cartilage barrier to reach subchondral bone. SCT@AHAMA microspheres is developed by combining Se‐CQDs with TPP and AHAMA. These micro/nano hydrogel microspheres, containing ultra‐small quantum dots, efficiently penetrate subchondral bone and regulate chondro‐bone metabolism, offering potential osteoarthritis treatment.

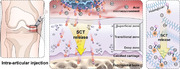

## 1 Introduction

Abnormal bone remodeling in the subchondral bone (SB) significantly induces articular cartilage degeneration, whose main pathological features include early bone loss via bone absorption, late subchondral sclerosis, and osteophyte formation via bone formation.^[^ ^1^ ^]^ Clinical interventions for SB exhibit superior therapeutic potential over cartilage treatment methods.^[^ ^2^, ^3^ ^]^ However, cartilage treatment does not fundamentally reverse the pathological process in SB during OA.^[^ ^2^ ^]^ Moreover, abnormal bone remodeling of SB intensifies the decomposition of the cartilage matrix.^[^ ^4^ ^]^ However, the highly anisotropic, dense, and avascular nature of the cartilage poses a challenge for drugs to overcome its steric hindrance and penetrate SB to exert therapeutic effects.^[^ ^5^, ^6^ ^]^ Therefore, the efficient penetration into SB and reshaping its bone metabolism by overcoming cartilage steric hindrance are key factors for OA treatment.

Under physiological conditions, SB maintains a low rate of osteogenesis with a few osteoclasts.^[^ ^7^ ^]^ Abnormal bone remodeling in SB caused by osteoclasts is an important pathological event that triggers OA.^[^ ^8^ ^]^ Articular cartilage lesions are formed by increased activity and numbers of osteoclasts in SB. However, the mechanism by which osteoclasts trigger cartilage degradation remains unclear. Several studies^[^ ^9^, ^10^ ^]^ have investigated the roles of osteoclasts in OA pathogenesis. Osteoclast precursor cells migrate to the cartilage layer, directly interacts with hypertrophic chondrocytes, to damage the articular cartilage matrix and osteochondral junction and further improve the release of numbers of growth factors such as the transforming growth factor beta 1 (TGF‐β1), insulin‐like growth factor 1 (IGF‐1), and platelet‐derived growth factor‐BB (PDGF‐BB), that interfere with cell metabolism by chondrocytes.^[^ ^11^ ^]^ In contrast, osteoclast‐induced H‐vessels formation may promote a transition from bone resorption to osteosclerosis in SB microenvironment in OA, accompanied by H‐vessel invasion into the cartilage and massive cytokine and inflammatory cell invasion into chondrocytes, which together disrupt its differentiated phenotype and metabolic homeostasis. Although the OA disease phenotype remains ambiguous, many animal and clinical trials have shown that targeting the early activation of osteoclasts can block the abnormal reconstruction of SB, thereby preventing articular cartilage degeneration.^[^ ^12^ ^]^

Mitochondrial reactive oxygen species (ROS), as secondary messengers, initiate the differentiation of hematopoietic stem cell or monocyte/macrophage progenitor cells into osteoclasts.^[^ ^13^ ^]^ Currently, Targeting mitochondria‐generated ROS effectively inhibits osteoclast activation.^[^ ^14^ ^]^ Abnormal ROS signaling is accompanied by a spatial and temporal distribution from the articular surface to SB. In vivo, several antioxidant molecules neutralize ROS and reduce oxidative stress.^[^ ^15^, ^16^ ^]^ It has also been demonstrated that scavenging excess ROS in the joints and inhibiting abnormal metabolism of cartilage and bone are essential for treating OA.^[^ ^16^ ^]^ However, most antioxidants are unable to efficiently go through the cartilage into SB due to the steric hindrance, which limits their curative efficacy.^[^ ^17^, ^18^ ^]^ Carbon quantum dots (CQDs) are 0D ultra‐small nanomaterials^[^ ^19^ ^]^ that can penetrate the dense structure of the cartilage to reach the chondrocytes, SB, and the medulla via intra‐articular injections.^[^ ^20^ ^]^ As a unique essential trace element, selenium plays important roles in several biological processes, for example, it exhibits antitumor, anti‐inflammatory, and antioxidant effects.^[^ ^21^ ^]^ Selenium‐doped CQDs (Se‐CQDs) perform redox regulatory functions and effectively scavenge ROS under oxidative stress, furthermore it possess superior optical attributes, such as enhanced fluorescence brightness and prolonged fluorescence duration, rendering them valuable for cellular imaging and tracking applications.^[^ ^22^ ^]^ However, During the development of OA, the capillaries within the joint cavity may undergo various changes, including capillary dilation and increased permeability, ultra‐small CQDs are easily removed and metabolized by capillaries and lymphatic vessels in the synovium^[^ ^23^ ^]^ which seriously affects their bioavailability and reduces their protective effects on the cartilage. Hence, an effective strategy of longer the residence time of biological agents within the joint cavity is important for OA therapy.^[^ ^24^, ^25^ ^]^

In this study, based on the anatomical concept of the osteochondral unit composed of hyaline cartilage, calcified cartilage, SB and inspired by the mechanism of OA development, primarily leverages the characteristics of quantum dots to enable their traversal through the cartilage layer, facilitating access to the subchondral bone region. This enables simultaneous regulation of cartilage‐bone metabolism for integrated arthritis treatment. we design and synthesis high‐permeability micro/nano hydrogel microspheres (SCT‐HA) using hyaluronic acid modified with aldehyde and methacrylic anhydride (AHAMA) and selenium‐doped carbon quantum dots grafted with triphenylphosphine (SCT) to regulate the osteoclasts activation in SB. SCT‐HA continuously released ultra‐small SCT after the Schiff base bond was broken in the weakly acidic environment of OA, which overcame the cartilage steric hindrance to reach SB and precisely regulated cartilage matrix synthesis and catabolism. In vitro, we evaluated the physical and chemical properties, SB permeation rate, mitochondrial targeting activity, and cartilage‐osteoclast metabolism of SCT‐HA microspheres. Then, we injected SCT‐HA microspheres into the joint cavity of OA mouse models to assess their ability to regulate cartilage metabolism and bone remodeling in SB, as well as to determine their action mechanisms. In conclusion, the high‐permeability SCT‐HA microspheres for SB osteoclasts targeting penetrated the cartilage by overcoming its steric hindrance via joint cavity injections, acted on SB, and reshaped the homeostatic balance in the OA microenvironment, suggesting their potential applications as efficient, rapid, and long‐acting ultra‐osmotic drug delivery systems for OA treatment.

## 2 Results and Discussion

### 2.1 Preparation of SCT‐HA for Bone/Chondrocyte Mitochondria

**Scheme 1:**
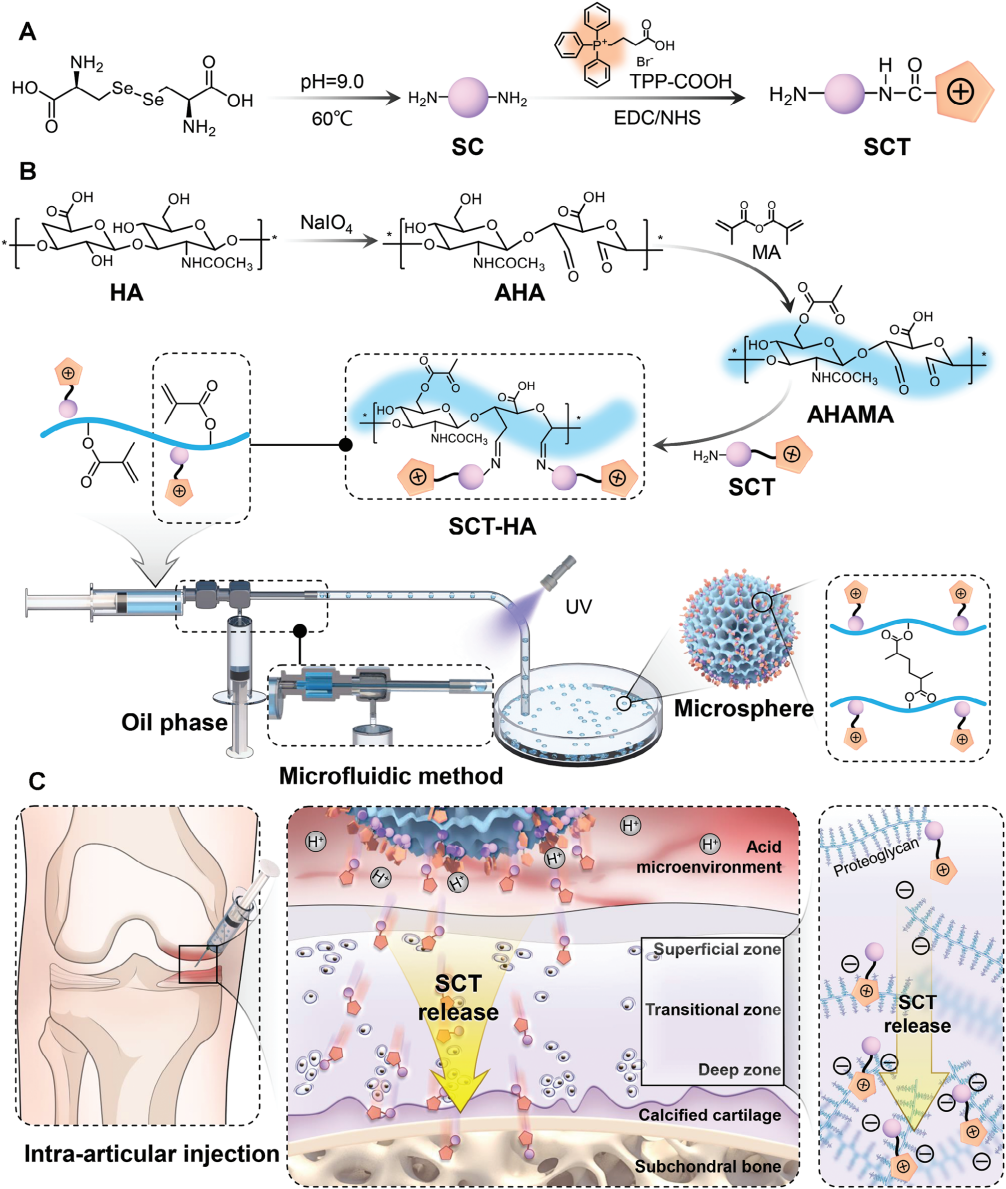
A) SCT synthesis. B) SCT‐HA preparation using microfluidic technology, which includes the synthesis process of AHAMA. C) SCT‐HA releases SCT continuously in a weakly acidic environment within the joint cavity, allowing it to penetrate the cartilage matrix and reach the SB for OA treatment. SC, SCT, HA, AHA, AHAMA and aha represent selenium‐doped carbon quantum dots, TPP‐doped SC, hyaluronic acid, hyaluronic acid modified with aldehyde and hyaluronic acid modified with aldehyde and methacrylic anhydride, respectively.

The preparation of micro/nano hydrogel microspheres included several key steps (**Scheme** **1**): SCT synthesis, AHAMA hydrogel preparation, and SCT‐HA microsphere preparation. First, SC was synthesized using L‐selenocystine under alkaline conditions through hydrothermal method. Selenium atoms in SC provide redox‐dependent reversible fluorescence and strong ROS scavenging abilities. Since intracellular ROS are mainly generated in mitochondria, we modified the surface of SC with TPP, a mitochondria‐targeting molecule, with excellent properties, to confer mitochondrial targeting and in situ ROS scavenging abilities to SC. SCT was obtained via an amide reaction between the COOH of TPP and the ‐NH_2_ of SC using 1‐ethyl‐(3‐dimethyl aminopropyl) carbodiimide/N‐hydroxysuccinimide(EDC/NHS) as the activator. Hydrogels are a unique scaffold material class that facilitates cartilage healing and mechanical function recovery. Hyaluronic acid (HA) is widely used in cartilage regeneration due to its excellent biocompatibility.^[^ ^26^, ^27^, ^28^ ^]^ Herein, we synthesized an aldehyde‐based and methacrylate‐modified injectable HA hydrogel (AHAMA). Sodium periodate was used to modify HA oxidatively, increasing the aldehyde groups in the material. These aldehyde groups can effectively bond to cartilage tissue for good tissue adhesion and form Schiff base bonds with the ‐NH_2_ of SC. These bonds can break under weak acid conditions associated with arthritis. The methacrylate‐modified HA exhibits inherent light‐curing properties, allowing for rapid gelation. Subsequently, we assembled a simple and effective microfluidic device to prepare SCT‐HA microspheres. The principle involved shearing the aqueous phase of SCT‐HA with a high flow rate liquid paraffin oil to form a water‐in‐oil structure, followed by AHAMA cross‐linking using ultraviolet (UV) light to stabilize the structure. Compared to conventional hydrogel biomaterials, hydrogel microspheres offer several advantages, including similarity to the natural extracellular matrix (ECM), strong workability, high water content and designability, and a large specific surface area, which enable the controlled delivery of various cells, drugs, and nanoparticles.^[^ ^29^, ^30^, ^31^, ^32^ ^]^

### 2.2 Characterization of the Physicochemical Properties and Cartilage Permeability of Highly Permeable Micro/Nano Hydrogel Microspheres Targeting Bone/Chondrocyte Mitochondria

**Figure 1:**
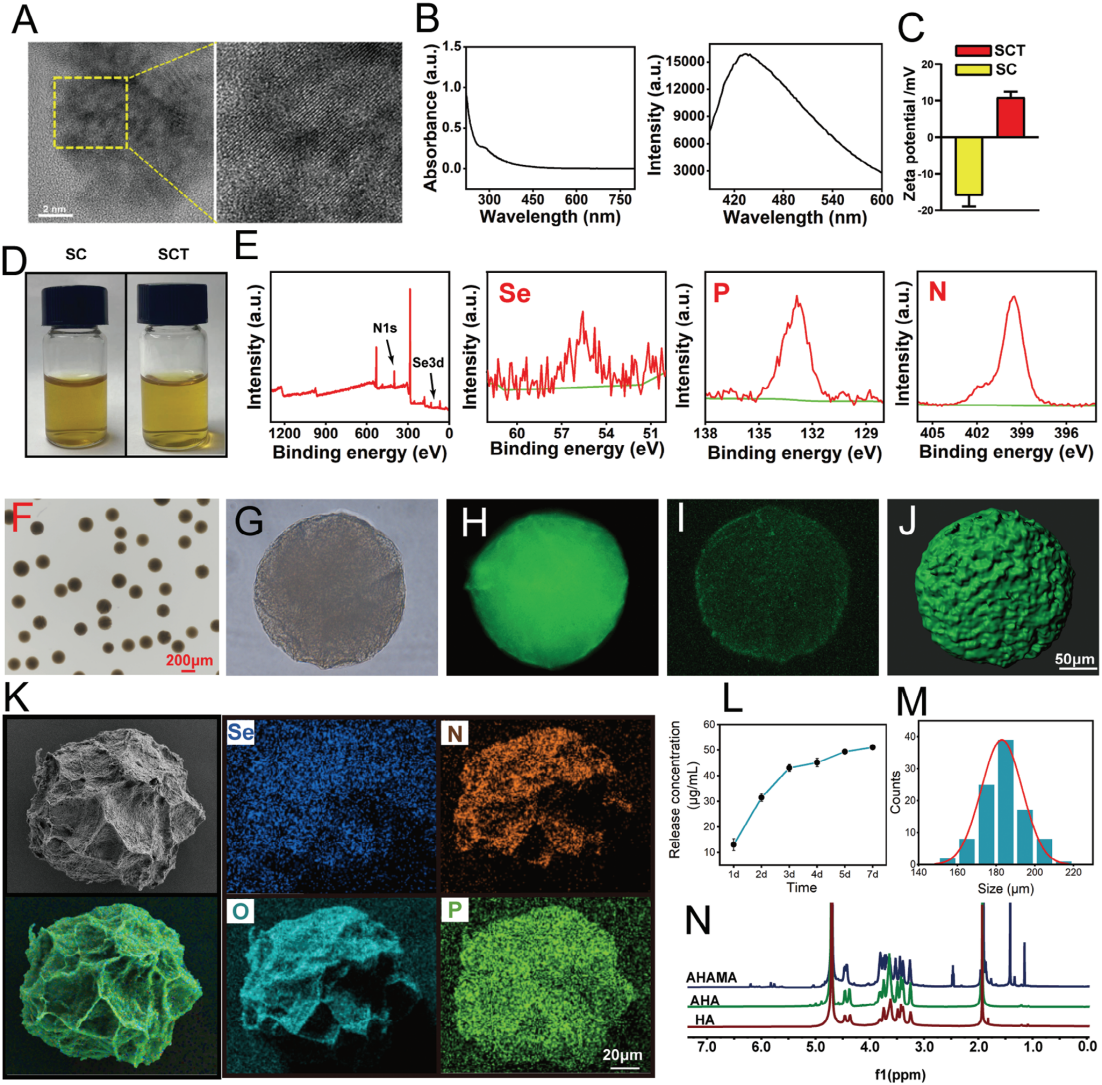
Preparation and characterization of highly permeable targeted bone/chondrocyte mitochondrial micro/nano hydrogel microspheres. A) TEM image of the SCT. B) UV absorption spectra and fluorescence spectra of the SCT. C) Zeta‐potential measurements of SC and SCT aqueous solutions. D) Photographs of SC and SCT in solution. E) Se, P, and N XPS spectra in the SCT. F) Light microscopy of SCT‐HA hydrogel microspheres prepared by microfluidic technology. G) Light micrographs of individual SCT‐HA hydrogel microspheres. H) Fluorescence micrographs of individual SCT‐HA hydrogel microspheres. I) Photographs of individual SCT‐HA hydrogel microspheres under laser confocal microscopy. J) 3D rendering of individual SCT‐HA hydrogel microspheres using Imris software. K) SEM images of SCT‐HA and elemental mapping. L) Release curves of SCT‐HA microspheres. M) Particle size distribution of microspheres. N) 1H NMR spectra of AHAMA, AHA, and HA was used to identify aldehyde and methacrylic anhydride groups.

Both particle size and surface properties play crucial roles in the capability of nanomaterials to infiltrate the cartilage matrix. Typically, smaller particles are more prone to penetration, whereas surface modifications can amplify or modify interactions with the matrix.^[^ ^5^ ^]^ The transmission electron microscope (TEM) results revealed the spherical shape of SCT with an average diameter of ≈5 nm (**Figure** **1**A; Figure S1, Supporting Information). The surface charges of SC and SCT were deter using the zeta potential (Figure 1C). The zeta potential of CQDs is −14.62 mV and SC was negatively charged with a potential of −16 mV, while the potential of SCT was +10.88 mV, again indicating that positively charged TPP is attached to the surface of SC (Figure 1C). Notably, the covalent attachment of the TPP cation to the SC surface did not significantly affect its morphology and size (Figures S1 and S2, Supporting Information). To examine the elemental composition of SCT and confirm the presence of Se and P, which represent a successful synthesis, we carried out the XPS (Figure 1E). The UV spectra of the SCT exhibited a characteristic peak at 260 nm. The photoluminescence results displayed a strong peak at 440 nm when excited at 350 nm (Figure 1B). Aldehyde‐based HAMA is an injectable adhesive hyaluronic acid (HA) hydrogel, which has been demonstrated in studies to possess robust mechanical strength, adhesion, and excellent biocompatibility. Additionally, it facilitates the seamless integration of the nascent bone layer with the native cartilage, thereby significantly enhancing cartilage regeneration.^[^ ^33^ ^]^ At 5.7 and 6.1 ppm, the ^1^H NMR spectrum of AHAMA displayed two newly emerged peaks corresponding to protons within the C═C bond of methacrylate (Figure 1N). For the joint cavity microenvironment, the 100 µm microsphere is not easy to be cleared by the joint cavity,^[^ ^31^ ^]^ the hydrogel microspheres exhibited an average particle size of 185 µm (Figure 1N) and could be uniformly dispersed in ultrapure water and injected using a syringe needle to meet the injection requirement (Figure 1F). SCT‐HA appeared green under general fluorescence and laser scanning confocal microscopy (LSCM), indicating the abundant presence of SCT (green) within the AHAMA microspheres (Figure 1G–J). Moreover, the scanning electron microscope (SEM) observations revealed numerous folds on the surface of the hydrogel microspheres, indicating their large specific surface area. Energy spectroscopy results confirmed the presence of O, N, P, and Se on the surface of hydrogel microspheres, providing further evidence for the successful SCT introduction (Figure 1K). SCT‐HA microspheres were placed in phosphate buffered saline (PBS) (pH = 6.8) at 37°C and Se ion concentration was detected by inductively coupled plasma emission spectrometer. SCT‐HA released a large amount of SCT in the first 3 days, and then the release rate slowed down. On day 7, the total release concentration and release rate of SCT were 5.11 µg mL^−1^ and 85.19%, respectively. (Figure 1L; Figure S3, Supporting Information). Further, we evaluated the degradation of AHAMA microspheres in a PBS solution (pH 6.8). The results showed that AHAMA microspheres degraded less in the first 3 weeks, the degradation rate of AHAMA microspheres increased after 3 weeks and the weight loss rate reached 61.38% at the 6th week (Figures S4 and S5, Supporting Information).

**Figure 2:**
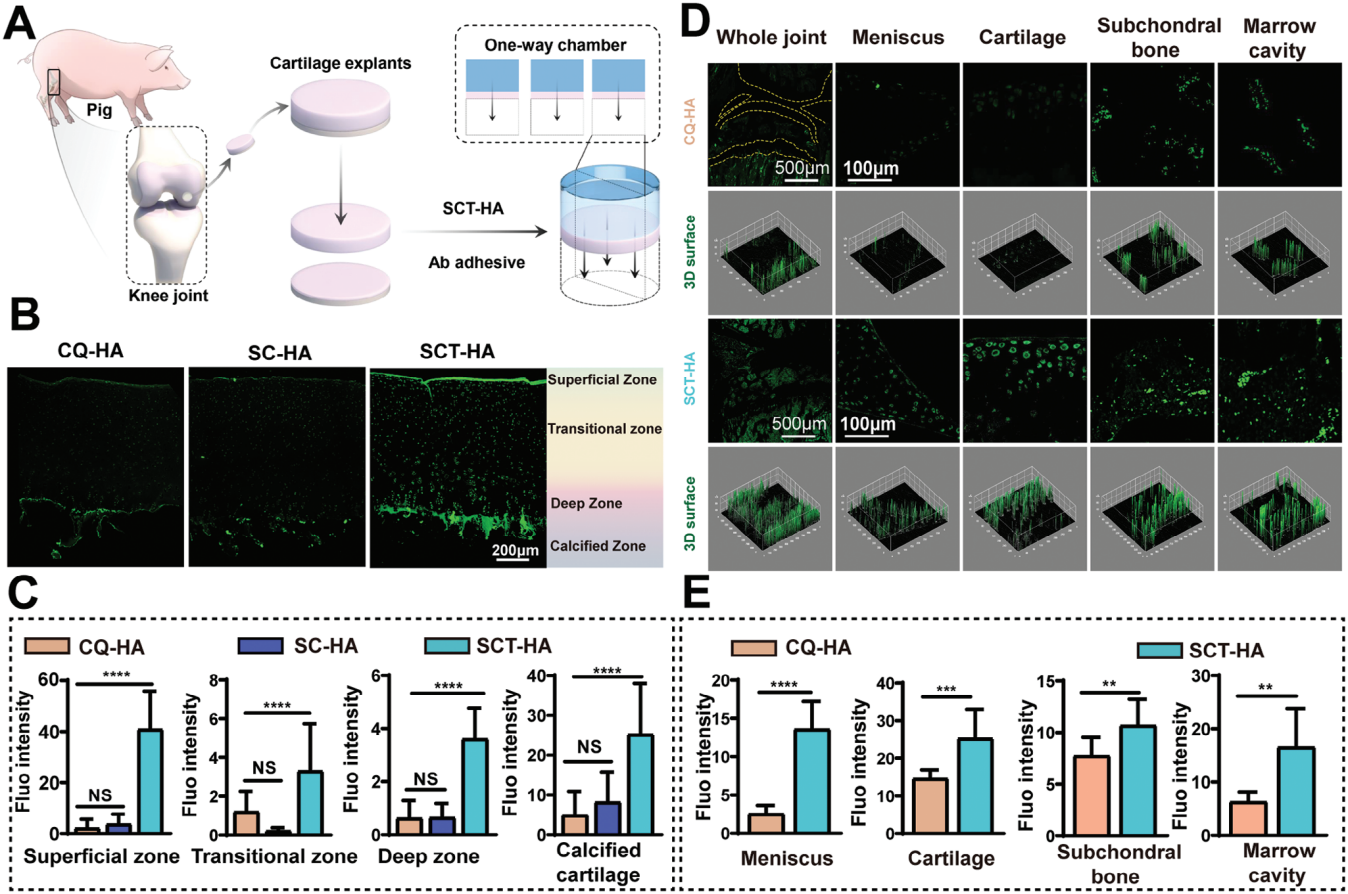
SB penetration of SCT‐HA in vitro and in vivo. A) Schematic illustration of the penetration assay of SCT‐HA in cartilage explants. B) Laser scanning confocal images of CQ‐HA, SC‐AHAMA (SC‐AH), and SCT‐HA penetrating articular cartilage explants. C) Fluorescence intensity analysis of the three materials penetrating the superficial zone, transitional zone, deep zone, and calcified cartilage of articular cartilage explants. n = 4 per group. D) Observation of the entire joint, meniscus, cartilage, SB, and bone marrow cavity in mouse knee sections after CQ‐HA and SCT‐HA injection into the joint cavity using laser scanning confocal observation and 3D surface reconstruction. E) Fluorescence intensity analysis of the meniscus, cartilage, SB, and marrow cavity in the knee joint of mice after CQ‐HA and SCT‐HA penetration. *n* = 4 per group. C) The data (mean ± standard deviation) were quantified using one‐way ANOVA followed by Tukey's posthoc multiple comparison test. N S: no significance. The p‐values < 0.05, 0.01, 0.001, and 0.0001 are presented as ^*^, ^**^, ^***^, and ^****^, respectively. E) The data (mean ± standard deviation) were quantified using Two‐tailed unpaired t‐test. NS: not significant. The p‐values < 0.05, 0.01, 0.001, and 0.0001 are presented as ^*^, ^**^, ^***^, and ^****^, respectively.

Furthermore, we evaluated the penetration characteristics of SCT‐HA into cartilage using fresh cartilage explants obtained from porcine joints. We observed deep penetration of SCT into cartilage, as evidenced by strong fluorescent signals throughout the entire cartilage layer, including the SB. Positively charged, ultra‐small nanostructured SCT can be released from AHAMA hydrogel microspheres to effectively cross the cartilage matrix under electrical charges. In contrast, the penetration range and ability of CQDs and SC during the same period significantly differed from SCT, and the strong fluorescence intensity observed on the cartilage surface is primarily attributed to the substantial retention of non‐penetrating quantum dot solution on the cartilage surface (**Figure** **2**A–C). In fact, we verified in vitro that SCT was indeed stronger than SC in penetrating porcine articular cartilage, which proved that SCT had a stronger advantage over SC in vivo. Thus, we propose that the combination of small size and positive charge of SCT enables more efficient cartilage penetration.

To investigate SCT penetration, we injected SCT‐HA and CQ‐HA into the knee joints of mice. After seven days, the mice were euthanized, and the knee cartilage specimens were examined using LSCM. We observed a noticeable green fluorescence in the knee treated with SCT‐HA, suggesting that SCT‐HA effectively prevents the abnormal bone remodeling process of subchondral bone. The SCT could penetrate the dense surface layer and reach cells in the deep cartilage zone, demonstrating greater penetration range and ability than CQ‐HA (Figure 2D,E). Furthermore, positive green signals were detected in the cells of the meniscus, SB, and bone marrow cavity, when SCT‐HA was injected into the joint cavity, the number of quantum dots was limited, and most of the quantum dots entered the subchondral bone during the long‐term degradation process, so they showed stronger fluorescence intensity than the cartilage surface (Figure 2D,E). It is important to highlight that OA affects both cartilage and SB, making the characteristics of SCT crucial for treatment. In traditional arthritis treatment strategies, a variety of nanomaterials have been developed to penetrate cartilage, including cationic micelles and inorganic nanoparticles. However, most materials are >20 nm in size and researchers have focused on cartilage rather than penetration of subchondral bone.^[^ ^2^, ^34^ ^]^

### 2.3 Highly Permeable Targeted Micro/Nano Hydrogel Microspheres Specifically Designed to Target Bone/Chondrocyte Mitochondria protect Chondrocytes

**Figure 3:**
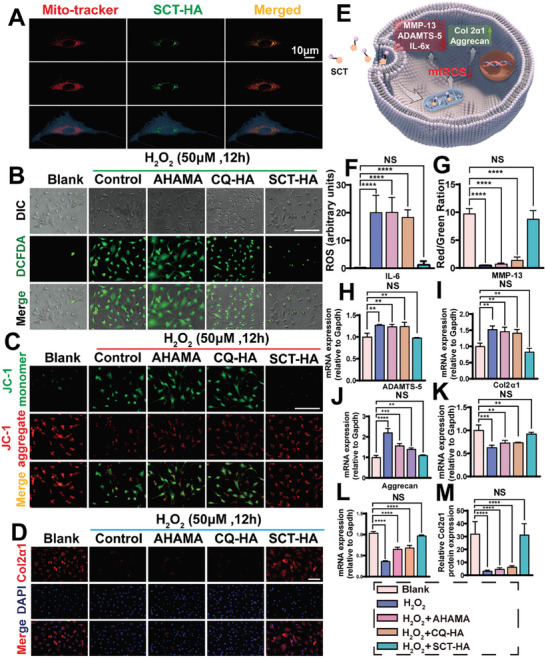
Protection of chondrocytes from H_2_O_2_‐induced ROS damage by highly permeable targeted bone/chondrocyte mitochondria micro/nano hydrogel microspheres. A) SCT‐HA targeting to the mitochondria in mouse chondrocytes: LSCM images showing Mito‐tracker mitochondrial dye (red), SCT autofluorescence (green), and merged (yellow). Mitochondria, SCT, and cell outlines (red, green, and blue) were reconstructed using Imris. B) Inhibition of H_2_O_2_‐induced ROS production by micro/nano hydrogel microspheres, DCFH‐DA staining of chondrocytes (green) indicating H_2_O_2_‐induced ROS production; scale bar = 100 µm. C) JC‐1 stain images of depolarized mitochondrial membranes after H_2_O_2_ intervention in chondrocytes. scale bar = 100 µm. D) Representative images showing the Col2α1 in chondrocytes treated with 50 µm H_2_O_2_ and co‐cultured with AHAMA, CQ‐HA, and SCT‐HA for 12 h; scale bar = 100 µm. E) SCT‐HA by targeting chondrocyte mitochondria inhibits ROS production, promotes cartilage anabolism, and inhibits chondrocyte catabolism and inflammation. F) Analysis of DCFH‐DA fluorescence intensity after H_2_O_2_ intervention in chondrocytes. *n* = 4 per group. G) Fluorescence intensity statistics of JC‐1 aggregate (red) and monomer (green) after H_2_O_2_ intervention in chondrocytes. *n* = 3 per group. H) IL‐6, I) MMP13, J) ADAMTS5, K) Col2α1, and L) Aggrecan expression in different groups after 12 h of treatment with 50 µM H_2_O_2_ on chondrocytes. *n* = 3 per group M) Immunofluorescence intensity statistics of the Col2α1 protein after H_2_O_2_ intervention in chondrocytes. *n* = 4 per group. F–M) The data (mean ± standard deviation) were quantified using one‐way ANOVA followed by Tukey's posthoc multiple comparison test. NS: no significance. The p‐values < 0.05, 0.01, 0.001, and 0.0001 are presented as ^*^, ^**^, ^***^, and ^****^, respectively.

To further evaluate the biosafety of AHAMA, CQ‐HA, and SCT‐HA on chondrocytes, we applied the cell counting kit‐8 (CCK‐8) and Live/Dead assays. These three groups exhibit similar amounts of live or dead cells at one and three days of culture (Figure S2A,C, Supporting Information). Similarly, no significant differences in the proliferative activity and viability of chondrocytes were detected among the groups at different time points (Figure S2D, Supporting Information). Additionally, to evaluate the mitochondrial targeting of SCT‐HA, chondrocyte mitochondria were labeled with a red fluorescent dye (Mito‐Tracker Deep Red FM) to take advantage of the autofluorescence characteristic of SCT. LSCM was used to observe the co‐localized (yellow) fluorescent signal of SCT and mitochondria, indicating that the TPP‐modified SC was efficiently localized in the cellular mitochondria (**Figure** **3**A). Overall, these results show that the SCT‐HA is biocompatible, and its loaded SCT has effective mitochondrial targeting to chondrocytes.

The mitochondria produce excessive ROS in chondrocytes affected by OA, but they can also be a sensitive target of ROS.^[^ ^13^ ^]^ In chondrocytes, ROS and oxidative stress can damage the mitochondrial DNA (mt‐DNA), leading to apoptosis, cellular aging, and matrix degradation, ultimately contributing to OA development. Next, we evaluated the distinct ROS scavenging abilities of different groups (Figure 3B). Compared to the H_2_O_2_, AHAMA, and CQ‐HA groups, the SCT‐HA group presented significantly reduced intracellular ROS, indicating a substantial anti‐ROS effect (Figure 3F). Moreover, we monitored the mitochondrial membrane potential (MMP) using JC‐1 to reveal early apoptosis of chondrocytes under ROS‐induced injury conditions. Normal MMP, early apoptosis, and depolarization of mitochondria were indicated by JC‐1 aggregates (red fluorescence) and monomers (green fluorescence), respectively. The articular chondrocytes MMP level of the SCT‐HA treated group was similar to the Control group (Figure 3C). SCT‐HA treatment could effectively protect the mitochondrial membrane and significantly inhibit the MMP decline and early apoptosis of chondrocytes under oxidative stress (Figure 3G).

Excessive oxidative stress and impaired mitochondrial function can induce damage, including imbalanced degradation and synthesis of the extracellular matrix, as well as inflammation in chondrocytes.^[^ ^35^ ^]^ Therefore, we used a Transwell chamber‐based co‐culture system of chondrocytes to assess the impact of SCT‐HA on inflammation and degeneration in chondrocytes by enhancing mitochondrial antioxidant function. Chondrocytes were exposed to H_2_O_2_ to simulate oxidative stress, and the protective effect of SCT‐HA on the inflammatory response and extracellular matrix catabolism in H_2_O_2_‐treated chondrocytes was assessed using qRT‐PCR (quantitative real‐time PCR) and immunofluorescence. A significant upregulation of IL‐6 (interleukin‐6), ADAMTs (a distintegrin and metalloproteinase with thrombospondin motifs), and MMP‐13 (matrix metalloproeinase‐13) and downregulation of Aggrecan and collagen type II alpha1 (Col2a1) were detected after 12 h of H_2_O_2_ treatment compared to the Control group (Figures 3H–K). However, the mRNA levels of groups treated with H_2_O_2_, AHAMA, and CQ‐HA microspheres did not differ. Nevertheless, the addition of SCT‐HA significantly downregulated ADAMTs, MMP‐13, and IL‐6 mRNA levels, while gradually upregulated Aggrecan and Col2a1, similar to the Blank group results. Col2a1, as a major component of the cartilage extracellular matrix, is widely recognized as a key marker of chondrogenic differentiation. After H_2_O_2_ intervention, chondrocytes were co‐cultured with AHAMA, CQ‐HA, and SCT‐HA. The immunofluorescence staining revealed markedly higher levels of Col2α1 protein staining in the H_2_O_2_‐free Blank group compared to the H_2_O_2_ group (Figure 3D), and the protein level of type II collagen (Col2a1) was significantly decreased after H_2_O_2_ treatment (Figure 3D–M). Meanwhile, SCT‐HA effectively restored Col2α1 protein levels compared to the H_2_O_2_ group (Figures 3D–M). These findings indicated that SCT‐HA exhibits a protective effect on chondrocytes to alleviate oxidative stress.

### 2.4 SCT‐HA Effectively Inhibits Osteoclast Differentiation and Function

Myeloid progenitor cells from the bone marrow differentiate into mononuclear macrophages, which fuse to form multinucleated giant cells known as osteoclasts.^[^ ^36^ ^]^ The macrophage colony‐stimulating factor (M‐CSF) and receptor activator of nuclear factor ligands (RANKL) closely regulated Osteoclast differentiation, When RANKL is not provided, precursor cells are unable to differentiate into osteoclasts in vitro. Through direct binding, RANKL effectively stimulates many signaling pathways, including NF‐κB (nuclear factor‐kappa B), MAPK (mitogen activated protein), and AKT (serine/threonine kinase). Additionally, the ROS level in cells can be effectively induced by RANKL, regulating osteoclast differentiation and maturation, significantly participating in osteoclast differentiation.^[^ ^37^ ^]^

**Figure 4:**
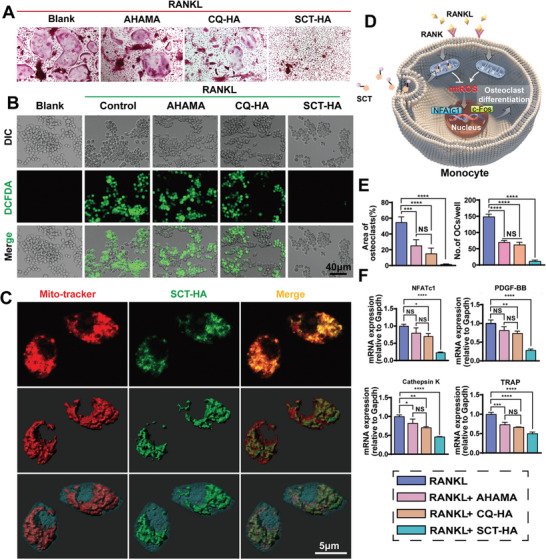
Inhibition of osteoclast differentiation and function through highly permeable targeted bone/chondrocyte mitochondrial micro/nano hydrogel microspheres. A) TRAP staining showing SCT‐HA inhibition of osteoclast differentiation induced by RANKL. B) SCT‐HA suppresses RANKL‐induced ROS production in RAW246.7 cells, as evidenced by the green DCFH‐DA staining. C) SCT‐HA specifically targets the mitochondria of RAW246.7 cells by laser confocal images of Mito‐tracker dye (red), SCT autofluorescence (green), and merged (yellow). The Imris reconstruction illustrates the mitochondrial dye, SCT, and cell outline (red, green, and blue). D) SCT‐HA targets mitochondria in mononuclear macrophages, leading to the inhibition of ROS production and suppression of osteoclast differentiation‐associated signaling pathways. E) Statistical analysis of TRAP staining in different treatment groups involved in osteoclast differentiation. *n* = 5 per group. F) Expression levels of osteoclast‐associated genes NFATc1, PDGF‐BB, Cathepsin K, and TRAP after three days of treatment in different subgroups. *n* = 3 per group. E‐F) The data (mean ± standard deviation) were quantified using one‐way ANOVA followed by Tukey's posthoc multiple comparison test. NS: no sgnificance. The p‐values < 0.05, 0.01, 0.001, and 0.0001 are presented as ^*^, ^**^, ^***^, and ^****^, respectively.

Thus, we conducted CCK‐8 and Live/Dead cell viability assays to evaluate the biosafety of AHAMA, CQ‐HA, and SCT‐HA on RAW246.7 cells (Figure S6A, Supporting Information). After one and three days of culture, all groups treated with AHAMA, CQ‐HA, or SCT‐HA exhibit almost the same proportion of dead and live cells (Figure S6B,C, Supporting Information). Furthermore, these groups presented the same proliferative activity and cell viability at different time points (Figure S6D, Supporting Information). Additionally, to assess the mitochondrial targeting of SCT‐HA, the mitochondria of RAW246.7 cells were labeled with a red fluorescent dye (Mito‐Tracker Deep Red FM) using the inherent autofluorescence of SCT. The co‐localized fluorescent signal (yellow) indicating the colocalization of SCT and RAW246.7 mitochondria was visualized usingl SCM. The results demonstrated the efficient localization of SCT in the mitochondria of RAW246.7 cells (**Figure** **4**C).

First, we investigated whether RANKL treatment induced detectable ROS production in RAW246.7 cells. Intracellular ROS production was evaluated using fluorescence microscopy and the cell‐permeable oxidation‐sensitive dye DCFH‐DA (dichlorodihydrofuorescein diacetate). The intracellular DCFH‐DA fluorescence intensity increased after 30 min of RANKL intervention (Figure 4B), suggesting that RANKL stimulated ROS production in RAW246.7 cells. There is no difference among RANKL, RANKL+AHAMA, and RANKL+CQ‐HA groups or between SCT‐HA and Blank groups in the intracellular ROS levels. Meanwhile, the intracellular ROS levels were significantly lower in the SCT‐HA group compared to the other three groups, showing an evident anti‐ROS effect (Figure S7, Supporting Information). Moreover, we established a co‐culture system using Transwell and mouse bone marrow‐derived macrophages (BMMs) to investigate the SCT‐HA intervention on osteoclast formation. After six days of RANKL induction, mouse BMMs cells exhibited increased size, pseudopod fusion, and pink cytoplasmic TRAP staining (Figure 4A), while the SCT‐HA group showed fewer TRAP‐positive cells (Figure 4E). RAW246.7 mouse macrophages serve as osteoclast precursor cells and can differentiate into mature osteoclasts upon RANKL stimulation. Notably, AHAMA showed a positive effect against osteoclast formation. The likely reason is that aldolylation modifications in AHAMA could potentially undergo schiff base bonding reactions with amino acids from RANKL and the inhibitory effect of HA on osteoclasts. Thus, we explored the impact of SCT‐HA on RANKL‐induced differentiation of RAW264.7 mouse macrophages into osteoclasts. Subsequently, we employed qRT‐PCR to analyze the key transcription factors involved in osteoclast differentiation, including the nuclear factor of activated T cells (NFATc1), platelet‐derived growth factor‐BB (PDGF‐BB), which specifically regulates H‐vessels, as well as osteoclast‐specific genes, such as anti‐tartrate acid phosphatase (TRAP) and histone proteinase K (cathepsin K). These factors were significantly downregulated in the SCT‐HA group, indicating that the internal loading of SCT in SCT‐HA can inhibit osteoclast differentiation and maturation (Figure 4F).

### 2.5 Gait Detection and Radiological Evaluation of SCT‐HA for OA Treatment

To discuss the therapeutic effects of SCT‐HA on OA, we established a mouse OA model via ACL (anterior cruciate ligament) transection and surgically induced joint mechanical instability.^[^ ^38^ ^]^ This model was used to assess the biological effects of the treatment. As shown in Figure S8 (Supporting Information), footprints from each experimental group were collected 10 weeks after surgery. Footprints were compared in Figure S8A (Supporting Information), with blue representing the healthy side and red representing the modeled side. Subsequently, the collected footprints were subjected to static parameter analysis. As depicted in Figure S8B–E (Supporting Information), a significant decrease in Footprints Stride Length was observed in the PBS group, while no difference was found in the SCT‐HA group compared to the Sham group. Additionally, in terms of Footprints Base of Supports, the PBS group exhibited a 35.87% increase compared to the Sham group, while the SCT‐HA group showed no significant difference. Subsequently, statistical analysis of foot length and toe spread revealed a 28.74% decrease and a 14.43% decrease, respectively, in the PBS group compared to the Sham group, while SCT‐HA treatment restored levels to those of the Sham group.

**Figure 5:**
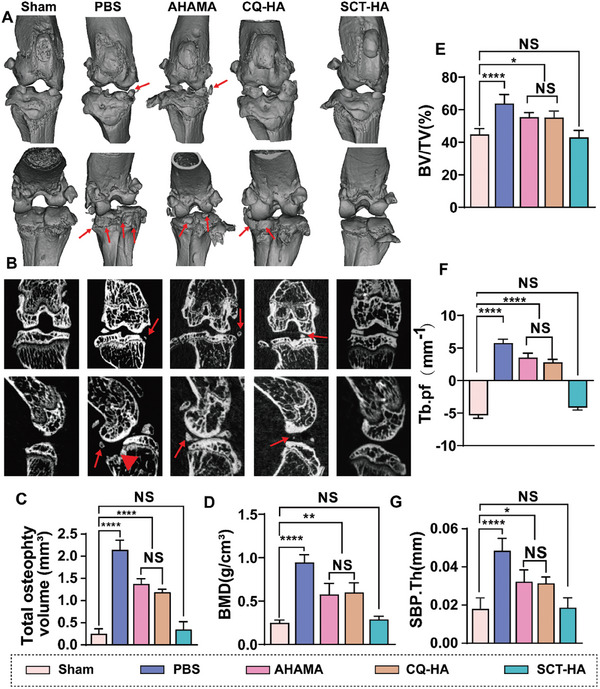
Radiographic evaluation of SCT‐HA for OA treatment. A) Representative anterior and posterior 3D reconstructed Micro‐CT images of the knee joint; red arrows indicate the presence of osteophytes. B) Representative coronal and sagittal 2D Micro‐CT images of the knee joint; red arrows indicate the presence of osteophyte s, and red triangles represent joint surface collapse. C) Statistical analysis of osteophytes volume. D) Statistical analysis of bone mineralization density (BMD) in the SB plate of each treatment group. Statistical analysis of E) Bone Volume Fraction (BV/TV), F) Trabecular Separation (Tb.pf), and G) SB Plate Thickness (SBP.Th) in each treatment group. C‐G) Data (mean ± standard deviation) were quantified from three independent experiments, one‐way ANOVA with a Tukey's posthoc multiple comparison test. The sham group were healthy mice, and the other groups were OA models. NS: no significance. The p‐values < 0.05, 0.01, 0.001, and 0.0001 are presented as ^*^, ^**^, ^***^, and ^****^, respectively.

To evaluate changes in the SB and knee joint, we euthanized the mice 10 weeks after the OA‐inducing surgery and analyzed it using micro‐CT. The reconstructed images showed decreased osteophyte volume in the AHAMA and CQ‐HAA groups compared to the group treated with PBS, which was still dramatically high compared to the Sham group. The SCT‐HA group exhibited improved joint morphology and lower osteophytes volume without statistical significance from the Sham group (**Figure** **5**A,C). Moreover, the SB revealed widening and dense trabeculae with a “fusion” phenomenon in the 2D images; some trabeculae were disorganized and unevenly arranged, resulting in a loss of meshwork. In the Sham and SCT‐HA group, the SB trabeculae were uniformly shaped, regular, and arranged more orderly (Figure 5B). Compared with the Sham group, the SB in PBS group exhibited higher bone mineralization density. However, the AHAMA and CQ‐HA groups showed decreased bone mineralization density compared to the PBS group but dramatically higher bone mineralization density than the Sham group. The quantitative results showed that the BV/TV (bone volume per tissue volume) values for the PBS and Sham groups were 63.81% and 44.85%, respectively. The AHAMA and CQ‐HA groups exhibited BV/TV values of 55.45% and 55.17%, respectively. In contrast, the SCT‐HA group had a BV/TV value of 42.94%, similar to the Sham group (Figure 5E). Additionally, the measured trend of the SB trabecular thickness (SBP.Th) in all five groups was consistent with the BV/TV results (Figure 4G). The trabecular pattern factor (Tb. Pf), which measures the degree of convex and concave trabecular surfaces, was −5.35 and 5.78 for the Sham and PBS groups, respectively. These values indicated increased bone remodeling activity in the osteoarthritic SB (BV/TV, SBP.Th). Nevertheless, the increased Tb.Pf suggested that the bone remodeling process was heterogeneous. The Tb.Pf did not differ between the SCT‐HA and Sham groups (Figure 5F), suggesting that SCT‐HA effectively prevents the abnormal bone remodeling process of subchondral bone. Although slowed OA progression was observed after AHAMA or CQ‐HA treatment, the micro/nano hydrogel microspheres exhibited the most pronounced therapeutic effect. In a mouse model of osteoarthritis, BV/TV is a vital parameter for quantifying the ratio of bone tissue volume to the total volume. It is typically expressed as a percentage, calculated by dividing bone volume by total volume. This ratio signifies the proportion of bone tissue within the total volume and serves as a crucial indicator for assessing bone density and structure. It can be employed to investigate the impact of skeletal disorders like osteoarthritis on mouse bone tissue. An elevation in BV/TV beneath the tibial joint signifies enhanced bone formation. Conversely, Tb.Pf is a parameter assessed in 3D space by contrasting alterations in solid volume and surface before and after pixel binarization. Generally, lower Tb.Pf values indicate a more densely interconnected trabecular structure, while higher values imply a more dispersed network structure. In certain instances, Tb.Pf values may turn negative, possibly due to the unique characteristics and distribution of bone structure, including the presence of numerous enclosed cavities and concave surfaces. This study observed that in a mouse model of osteoarthritis, the increase in BV/TV beneath the tibial joint is associated with an elevation in Tb.Pf, implying that arthritis may result in uneven bone formation beneath the cartilage.

### 2.6 SCT‐HA Protects Cartilage and Inhibits Abnormal SB Remodeling

**Figure 6:**
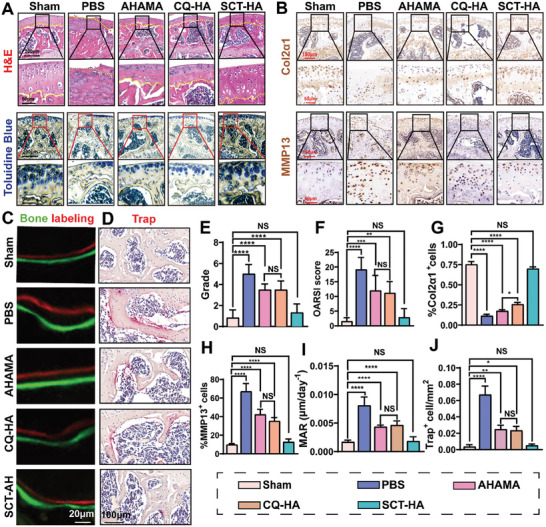
The SCT‐HA inhibits abnormal bone remodeling in the SB and prevents cartilage degradation. A) Representative images of toluidine blue and H&E staining. B) Col2α1 and MMP13 levels by immunohistochemical staining. C) Calcein green and Alizarin red double labeling using fluorescence microscopy. D) Representative TRAP‐stained SB sections of the tibia. E) Grade and F) OARSI scores. n = 6 per group. G) Summarized results of relative Col2α1 expression. *n* = 6 per group. H) MAR quantification. n = 5 per group. J) Quantification of TRAP^+^ multinuclear cells. *n* = 6 per group. E–J) Data (mean ± standard deviation) were quantified from three independent experiments, one‐way ANOVA with a Tukey's posthoc multiple comparison test. NS: no significance. The p‐values < 0.05, 0.01, 0.001, and 0.0001 are presented as ^*^, ^**^, ^***^, and ^****^, respectively.

Within cartilage, chondrocytes, the sole cell type responsible for cartilage matrix secretion, maintenance of cartilage organization, response to growth factors, and participation in cartilage repair, among other functions, have garnered the most significant focus.^[^ ^39^ ^]^ Within subchondral bone, abnormally activated osteoclasts instigate pathological alterations in early osteoarthritis, affecting both the bone cortex beneath the cartilage (referred to as “ivorying”) and the bone's marginal area (known as “bone spurs”), which are regarded as indicative of osteoarthritis. Thus, osteoarthritis should be perceived as a disorder involving abnormal cartilage‐bone metabolism, rather than merely a cartilage issue.^[^ ^2^ ^]^ Next, we conducted various tests, including H&E staining, special staining, immunohistochemical testing, and fluorescent double‐labeling, on knee specimens collected 10 weeks after the operation to explore the impact of SCT‐HA on the comprehensive treatment of SB remodeling and articular cartilage. The H&E and toluidine blue staining demonstrated that cracks and deformation were common in the PBS group, the two most severe articular cartilage erosions. The AHAMA and CQ‐HA groups also exhibited significant erosion, while reduced damage and better morphological integrity of the cartilage surface were found in the SCT‐HA group, resembling more the Sham group (**Figure** **6**A). Subsequently, we evaluated OA severity using the OARSI scale. The OARSI scores were reduced by 37.5% in the AHAMA group and 41.7% in the CQ‐HA group compared to the PBS group. The SCT‐HA group exhibited the highest reduction, with OARSI scores decreasing by 85.2% (Figures 6E,F). These results suggested that SCT‐HA microspheres effectively preserved the thickness of articular cartilage. Additionally, we evaluated the levels of two crucial proteins in the synthesis and catabolism of articular cartilage: Col2α1 and MMP13 (Figure 6B). Compared to the Sham group, the groups treated with PBS, AHAMA, and CQ‐HA presented significantly lower Col2α1 levels. Meanwhile, the Sham and SCT‐HA groups did not differ (Figure 6H). MMP13 (matrix metalloproteinase 13) plays distinct but interrelated important roles in cartilage and subchondral bone. MMP13 expression in cartilage is often associated with cartilage degradation and destruction. In diseases such as osteoarthritis, continuous oxidative stress environment (ROS) leads to cartilage damage, and the expression of MMP13 is significantly upregulated, which then decomposes collagen in cartilage, leading to the degeneration and loss of normal structure of cartilage tissue. Therefore, the expression of MMP13 can reflect the level of oxidative stress and inflammation in osteoarthritis to a certain extent.^[^ ^40^ ^]^ The expression level of MMP13 was significantly elevated in the PBS, AHAMA, and CQ‐HA groups compared to the Sham group. Nevertheless, the SCT‐HA and Sham groups did not differ (Figure 6G). These findings suggested that SCT‐HA microspheres can target and attach to the damaged cartilage surface, releasing SCT to go through the cartilage matrix and enhancing the delivery efficiency of biologics to the cartilage. Moreover, the positively charged ultra‐small SCT structure enables it to penetrate the cartilage and selectively target chondrocytes within the cartilage matrix, effectively safeguarding chondrocytes from peroxidative damage and reducing in situ chondrocyte ROS production, thereby protecting both chondrocytes and the cartilage matrix. Overall, this promising approach provides an appealing strategy for OA treatment.

Abnormal SB remodeling is a pathological characteristic of OA. Excessive activation of osteoclasts initiates the abnormal bone remodeling process in OA. Hence, inhibiting osteoclast activity can effectively alleviate SB pathology. We employed histological anti‐tartrate acid phosphatase (TRAP) staining to visualize osteoclasts in the subchondral bone. The TRAP staining revealed a significant increase in SB osteoclasts in the PBS group compared to the Sham group, indicating abnormal osteoclast activation in the SB under OA conditions. In ACLT mice treated with AHAMA and CQ‐HA, the number of high TRAP^+^ osteoclasts was reduced. However, only SCT‐HA restored the abnormal TRAP^+^ osteoclasts to the Sham group levels (Figure 6D,J). Abnormal activation of osteoclasts can lead to abnormal SB metabolism, MAR indirectly assesses the level of osteoclast activity. Thus, we assessed the MAR using fluorescent double‐labeling in the SB. PBS mice exhibited increased SB formation compared to the Sham group (green and red), while AHAMA and CQ‐HA treatment reduced the abnormal SB formation (Figure 6C,I). SCT‐HA treatment restored the MAR to the level of the Sham group. In summary, these results indicated that SCT‐HA could comprehensively improve both articular cartilage and SB abnormal metabolism, providing an integrated and comprehensive therapeutic effect on arthritis.

### 2.7 SCT‐HA Inhibits Angiogenesis in SB

**Figure 7:**
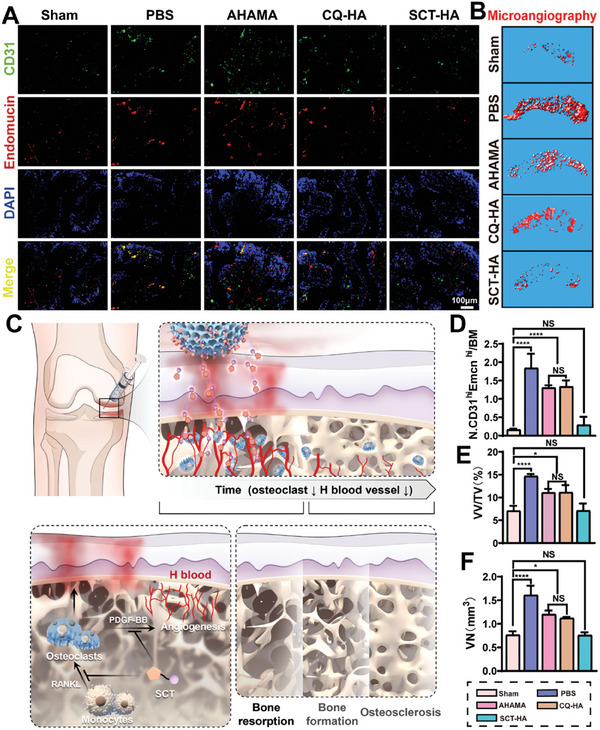
The SCT‐HA inhibits blood vessel formation in the SB. A) Immunofluorescence images of H‐vessels (CD31^hi^Emcn^hi^) in SB of each treatment group. B) Microfil MV‐122 angiography of the SB in each treatment group after 3D reconstruction with Micro‐CT. C) Schematic diagram illustrating the inhibition of abnormal SB remodeling by SCT‐HA. SCT‐HA inhibits the differentiation of monocyte macrophages into osteoclasts and PDGF‐BB production to suppress the abnormal invasion of SB blood vessels in OA. By inhibiting blood vessels, which act as the “executors” of abnormal bone remodeling, SCT‐HA also prevents the occurrence of abnormal bone remodeling in SB. D) Quantitative statistical analysis on the H‐vessels within the SB of each group. *n* = 6 per group. E) Vascular volume relative to the tissue volume (VV/TV) in the SB. *n* = 3 per group. F) Vessel number (VN). n = 3 per group. D‐F) Data (mean ± standard deviation) were quantified from three independent experiments, one‐way ANOVA with a Tukey's posthoc multiple comparison test. NS: no significance. The p‐values < 0.05, 0.01, 0.001, and 0.0001 are presented as ^*^, ^**^, ^***^, and ^****^, respectively.

The bone is a highly vascularized tissue, angiogenesis is one of the critical factors in the development and symptomatic progression of osteoarthritis, any factor that promotes angiogenesis could serve as a potential target for osteoarthritis treatment. Therefore, inhibiting angiogenesis has become a potent target in the treatment of OA.^[^ ^41^ ^]^ Hence, we explored the impact of SCT‐HA on SB angiogenesis. Using Micro‐CT‐based angiography, we observed a significant increase in the number (VN) and volume (VV/TV) of vessels in the SB of mice in the PBS group compared to the Sham group. Additionally, the SCT‐HA group suppressed the increasing number (VN) and volume (VV/TV) of vessels in the SB compared to AHAMA and CQ‐HAA groups, presenting values similar to the Sham group (**Figure** **7**B,E,F).

Bone intramedullary capillaries is able to be classified into two distinct subtypes, H‐type and L‐type, via dual labeling of bone vascular endothelial cells. H‐type vascular endothelial cells exhibit dramatically high Emcn and CD31 levels, mainly in the bone metaphysis and endosteum.^[^ ^42^ ^]^ They form a columnar distribution and are interconnected by vascular loops. Osteoprogenitor cells selectively distribute around H‐type blood vessels. In contrast, low Emcn and CD31 levels are observed in L‐type capillary endothelial cells. They are primarily found in the bone diaphysis as sinusoidal capillaries, surrounded by abundant infiltration of hematopoietic cells. The number of osteogenic progenitor cells associated with L‐type vessels is extremely low.^[^ ^42^ ^]^ H‐type promotes increased bone mass, accelerates bone formation, and coupling with osteogenesis. In osteoarthritis patients, H‐vessels originating from the subchondral bone infiltrate the tide line and extend into the articular cartilage via vertical microchannels. This means that signaling substances secreted by chondrocytes and osteoclasts can be transported and diffused through these interconnected vessels.^[^ ^1^ ^]^ Consequently, the involvement of H‐vessels in cartilage and subchondral bone represents a temporally distinct yet interconnected process. Thus, we analyzed the vascular types inhibited by SCT‐HA using dual immunofluorescent staining of CD31 and Emcn in the SB. In the SB of PBS‐treated mice subjected to AHAMA and CQ‐HA, the number of CD31^hi^Emcn^hi^ vessels significantly decreased compared to the PBS group. However, only SCT‐HA restored the CD31^hi^Emcn^hi^ vessels similar to the Sham‐operated control group (Figure 7A,D). The close spatiotemporal relationship between bone growth and blood vessel formation is known as “vascular‐bone coupling”.^[^ ^43^ ^]^ This coupling is achieved by H‐type vessels induced by osteoclasts producing PDGF‐BB, which can alter the differentiation, proliferation of surrounding osteogenic progenitor cells, actively promote bone formation.^[^ ^44^ ^]^ SCT‐HA inhibited CD31^hi^Emcn^hi^ vessels in the SB of OA, suppressing the “executors” of abnormal SB remodeling (Figure 7C).

### 2.8 Transcriptional Profiling of SCT‐HA and Regulation of SB Function

**Figure 8:**
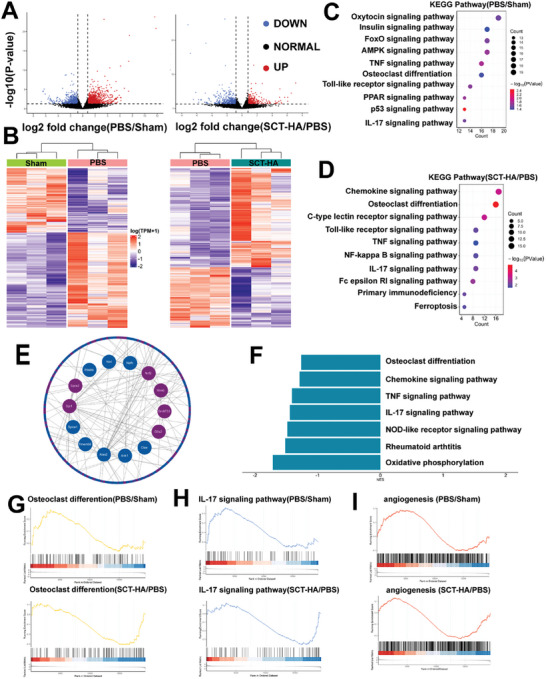
Transcriptional profiling of SCT‐HA regulating SB function on mouse OA model. A) Volcano plots for PBS versus Sham and SCT‐HA versus PBS group comparisons. Upregulated genes are marked red, and downregulated genes are marked blue. B) Heatmap illustrating the differential gene expression for PBS versus Sham and SCT‐HA versus PBS groups. Functional enrichment analysis of KEGG pathways using differentially expressed genes (DEGs) between C) PBS versus Sham groups D) SCT‐HA versus PBS groups. E) Alterations in osteoclast activation‐related genes between the SCT‐HA and PBS groups. Blue indicates downregulated osteoclast‐related genes, while purple indicates upregulated osteoclast‐related genes. F) GSEA of KEGG pathways between SCT‐HA and PBS groups. Normalized enrichment scores (NES) represent the combined dataset of KEGG pathway gene sets. (G, H, I) GSEA after applying a threshold screening.

Knee specimens were isolated from mice 10 weeks postoperatively, and RNA‐seq was carried out in the SB of Sham, PBS, and SCT‐HA groups (**Figure** **8**A). These results demonstrated alterations in osteoclastic differentiation‐related signaling pathways in the PBS group, such as AMPK (adenosine monophosphate activated protein kinase), TNF (tumor necrosis factor), IL‐17 (interleukin‐17), and p53 (protein 53) signaling pathways, compared to the Sham group (Figure 8A). These findings indicated a notable increase in the inflammatory response and osteoclastic activation in the PBS group (Figure 8B,C). The Gene Set Enrichment Analysis (GSEA) revealed the downregulation of osteoclast‐related genes, the IL‐17 signaling pathway, and the angiogenic signaling pathway (Figures 8G–I). The signaling pathways altered in the SCT‐HA group compared to the PBS group included the IL‐17 signaling pathway, NF‐KB signaling pathway, and pathways associated with osteoclastic and inflammatory responses, such as iron death (Figure 8D). Given osteoclast‐associated genes, we investigated those that underwent significant changes in the SCT‐HA group and observed substantial downregulation of most of them (Figure 8E). To gain further insight into the alterations of individual signaling pathways in the SCT‐HA group compared to the PBS group, we analyzed the changes in weighted scores for each signaling pathway using GSEA. The findings indicated the downregulation of all inflammation‐related signaling pathways, including angiogenic, IL‐17, and osteoclastic differentiation signaling pathways (Figure 8F). Therefore, the alleviated angiogenic, inflammatory, and osteoclast‐related signaling pathways in the SCT‐HA group demonstrated the anti‐inflammatory, osteoclast‐inhibiting, and angiogenic functions of this material. Overall, a single arthritis treatment strategy cannot fundamentally delay the progression of osteoarthritis, and regulating chondro‐bone metabolism will provide stronger adaptability for arthritis treatment. Selenium quantum dots act on cartilage and subchondral bone with superpermeability and maintain chondro‐bone metabolic balance, but the detailed mechanism still needs further study. In addition, the fluorescence properties of quantum dots will provide a new option for the integrated treatment of osteoarthritis.

## 3 Conclusion

In summary, the SCT‐HA hydrogel microspheres anchored to the damaged cartilage surface through various mechanisms, including dynamic Schiff base reactions forming imine bonds, hydrogen bonds, and physical interpenetration. Moreover, the SCT‐HA hydrogel microspheres responded to the weak acidic environment by releasing SCT to penetrate the cartilage and reach the SB, inhibiting peroxidative damage to chondrocytes and reducing the differentiation and maturation of mononuclear macrophages into osteoclast. The effects of SCT on chondrocytes were further validated in a mouse OA model. The released SCT after SCT‐HA injection efficiently penetrated the SB and significantly inhibited abnormal cartilage metabolism and SB remodeling, decelerating the osteoarthritic process. The injectable SCT‐HA microspheres presented exceptional SB penetration properties, comprising a novel SB intervention approach and new prospects in arthritis treatment.

## 4 Experimental Section

### SCT Synthesis

Selenium‐carbon quantum dots (Se‐CQDs, SC) were synthesized using a hydrothermal method.^[^ ^45^ ^]^ A water‐based dispersion was formed by mixing L‐selenocysteine (0.3 g) with 35 mL of water. The pH was adjusted to 10 using 0.1 m sodium hydroxide. Then, under nitrogen protection, a 20‐h heating of the reaction mixture was at 60 °C. After the reaction, the brown solution was recovered by 10‐min centrifugation at 12 000 rpm, and the supernatant was retained for dialysis. TPP (2.0 mL, 3.5 mm) was activated using 1‐ethyl‐(3‐dimethyl aminopropyl) carbodiimide/N‐hydroxysuccinimide (EDC/NHS) as an activator within 3 h. To produce TPP‐functionalized Se‐CDs (SCT), a 12 h reaction of the activated TPP with Se‐CDs was conducted. Excess TPP was removed by dialysis.

### Preparation of AHAMA Hydrogels

Aldehyde‐based hyaluronic acid (AHA) was synthesized as previously described.^[^ ^33^ ^]^ Briefly, after completely dissolving hyaluronic acid (HA, 1 g) in water (100 mL), 5 mL sodium periodate (0.5 m) was added dropwise. Next, a 2 h stirring of the mixture was carried out under light protection at room temperature (RT). To deactivate the unreacted periodate, a reaction period of 1 h was allowed following the addition of 1 mL of ethylene glycol. The reaction product was dialyzed for three days and then freeze‐dried to obtain AHA.

The double bond modified AHA synthesis was performed as previously described using methacrylate for the modification. Briefly, after completely dissolving hyaluronic acid (HA, 1 g) in water (100 mL), 1 mL methacrylic anhydride was added. Then, the mixture was maintained at pH 8–8.5 for 12 h. The entire process was performed under ice‐cold conditions. After the reaction, the solution was dialyzed for two days, then lyophilized to obtain AHAMA. Quantitative ^1^H NMR was used to determine the percentage of double bonds by dissolving the sample in D_2_O. The AHAMA aldehyde content was determined using a solution of tert‐butylammonium diazoate and trinitrobenzene sulfonic acid (TNBS).

### Preparation of Highly Permeable Targeted Bone/Chondrocyte Mitochondrial Micro/Nano Hydrogel Microspheres (SCT‐HA)

The SCT‐HA was prepared using a laboratory‐made microfluidic device.^[^ ^46^ ^]^ In the aqueous phase, SCT was mixed with a hydrogel solution containing AHAMA, SCT, and a photoinitiator. Meanwhile, Span 80 and paraffin oil were mixed in the oil phase, and this mixture was injected into the microfluidic device inlet using a syringe to control the flow rate between aqueous and oil phase.^[^ ^47^ ^]^ After freezing at −30 °C, the droplets were cross‐linked using UV light to form SCT‐HA. Finally, the liquid paraffin was removed by ether, and the microspheres were suspended in water, freeze‐dried, and stored.^[^ ^48^, ^49^ ^]^

### Characterization of SCT and SCT‐HA Microspheres

The SCT and SCT‐HA microspheres were characterized as follows: i) Transmission electron microscopy (TEM, FEI TF20, USA) at an accelerating voltage of 200 kV (2100F) was used to characterize SCT morphology. ii) The zeta potential and size distribution of SCT particles were characterized by a nanoparticle size potentiostat (NanoBrook 90plus PALS, Brookhaven, USA). iii) UV–vis absorption experiments were conducted using a UV spectrophotometer (UH5300) in a 1 cm quartz cell. The fluorescence spectrum of SCT was measured using an F‐4600 fluorescence spectrophotometer. iv) X‐ray photoelectron spectroscopy (XPS) detected the elemental valence and content of SCT. v) The wet morphology and autofluorescence of SCT‐HA microspheres were observed using a microscope (LSM800, ZEISS, Germany), and the particle size distribution of 100 SCT‐HA microspheres was measured using ImageJ. vi) The dry morphology of SCT‐HA microspheres was observed using scanning electron microscopy combined with energy spectroscopy to verify the elemental distribution (O, N, Se, and P). vii) Laser scanning confocal microscopy (LSCM) (ZEISS) was utilized to observe the autofluorescence luminescence of SCT in microspheres. viii) To quantify the release of SCT from the SCT‐HA microspheres, SCT‐HA microspheres were first immersed in a weakly acidic phosphate buffer (pH 6.8) and agitated using a shaker at 80 rpm. Then, the supernatant was replaced with PBS once every 2 days. The collected supernatant was analyzed separately for Se content using inductively coupled plasma emission spectrometer (iCAP7600, Thermo, USA). The degradation of SCT was performed in a PBS solution (pH 6.8). First, the study weighed 12 mg SCT microspheres (M_0_) and added 20 mL PBS solution, which was changed every three days. The supernatant solution was removed at 1w, 3w, and 6w respectively, the undegraded microspheres were retained and lyophilized, and their weight (M_1_) was recorded. The formula of degradation rate is (M_0_‐M_1_) /M_1_ ×100%.

### Primary Chondrocyte Isolation

To isolate mouse primary chondrocytes (MCCs), the tibial plateau, femoral condyle, and femoral head of mice were collected, and the cartilage fragments were used to isolate the MCCs. After cutting into small pieces and 30 min digestion using 0.25% trypsin at 37 °C, and washing using PBS, 8‐h digestion was conducted at 37 °C with 0.25% collagenase II. Next, the MCCs were collected by filtration using a 70 µm cell filter and 5‐min centrifugation at 1000 rpm. Double antibodies (1%)‐ and FBS (10%)‐contained DMEM medium was used to culture these isolated cells. Finally, 3rd generation chondrocytes were used for subsequent experiments.

### Mitochondrial Targeting by SCT‐HA Microspheres

Furthermore, the SCT‐HA microspheres were tested for SCT release on primary chondrocytes and mitochondrial targeting on Raw264.7 cells. Briefly, after 4 h incubation in the 10% FBS‐contained DMEM medium and washing, replacement of the medium was carried out. Then, cells were incubated for 4 h with SCT‐HA microspheres followed by 4 h staining using 0.5 µM Mito‐Tracker to analyze mitochondrial co‐localization. Staining was performed using Deep Red FM (Biyuntian, China). Finally, LSCM was performed using a ZEISS Axio Imager M1 microscope (Germany). The same method was employed to observe mitochondrial targeting in Raw264.7 cells.

### Cartilage Penetration

Porcine articular cartilage could be useful for certain aspects of OA research and for validating penetration experiments.^[^ ^50^ ^]^ Articular cartilage was obtained from the patellar groove of the femur using a 4 mm diameter ring drill bit. The extracted articular cartilage was washed with sterilized PBS. The penetration test was conducted using a self‐designed unidirectional diffusion chamber (Figure 2A). The articular cartilage explant was enclosed within the chamber, divided into upper and lower layers. The lower layer and cartilage periphery were filled with Ab adhesive, which is a commercially available glue to ensure that the quantum dots could only penetrate from the top down, while the upper layer contained SCT‐HA microspheres soaked in PBS, positioned on the cartilage surface. After 48 h, a 1 mm thick section was excised from the center of the cartilage and promptly observed using laser confocal microscopy (LSCM) (ZEISS, Axio Imager M1, Germany).

Moreover, the in vivo cartilage penetration experiments were conducted on 10‐week‐old male wild‐type C57BL/6 mice. After surgery in the OA model (refer to the OA animal model section for detailed information), the joint cavity was injected with SCT‐HA microspheres. After seven days, the knee joints of mice were sectioned and observed under a laser confocal microscope (LSCM) (ZEISS).

### Cell Biocompatibility

To assess the impact of AHAMA, CQDs@AHAMA (CQ‐HA), and SCT‐HA on the proliferation of primary chondrocytes, live/dead staining was carried out. Briefly, in a 24‐well transwell plate, the low chamber was added with 1.0×10^4^/mL mouse primary chondrocytes (MCCs) and the up chamber with AHAMA, CQ‐HA, and SCT‐HA. Then, cells were incubated with the CalceinAM/PI assay working solution (250 µL) (Biyuntian) for 30 min and visualized using a microscope after one or three days of culture.

The cell proliferation was also measured after incubation with AHAMA, CQ‐HA, and SCT‐HA. Briefly, after 1‐ or 3‐day incubation in a 96‐well transwell plate, addition of CCK‐8 solution (10 µL) to each well and 1 h incubation at 37 °C were carried out. Next, a FlexStaston3 enzyme labeler (Molecular Devices, Japan) was used to detect and record the absorbance values at 450 nm. A similar methodology was applied to assess the effects of AHAMA, CQ‐HA, and SCT‐HA on the proliferation of Raw264.7 cells. The effective concentration of quantum dots was 5 µg mL^−1^ in vitro.

### Measurement of Intracellular ROS and Mitochondrial Membrane Potential

First, MCCs were inoculated in 6‐well plates and cultured with H_2_O_2_ to induce oxidative stress. Cells were then co‐cultured with different subgroups (PBS, AHAMA, CQ‐HA, and SCT‐HA) for 12 h. Afterward, all cells were treated with the fluorescent dye DCFH‐DA (10 µm) (DCFH‐DA, Beyotime, China). Cells were washed three times and observed under a microscope (LSM800, ZEISS, Germany). After 12 h of the same treatment, cells were stained with JC‐1 (Beyotime) for 30 min and observed using an LSM800 microscope. Similar methods were employed to evaluate the effects of AHAMA, CQ‐HA, and SCT‐HA on ROS in Raw264.7 cells.

### OA Model‐Related Gene and Protein Assays

To assess the roles of SCT‐HA micro‐hydrogel in OA after mitochondrial ROS modulation, MCCs were first treated with H_2_O_2_ (50 µm) and exposed to different treatments, including PBS, AHAMA, CQ‐HA, and SCT‐HA, for 12 h. Total RNA was extracted from MCCs and subjected to reverse transcription, followed by RT‐qPCR. Primer sequences for Aggrecan, Col2α1, MMP‐13, ADATMS‐5, and IL‐6 are provided in Table S1 (Supporting Information). This experiment was repeated three times. After 12 h of treatment, chondrocyte Col2α1 levels were assessed via immunofluorescence staining. After twice washing with PBS and 10 min fixation using 4% paraformaldehyde, 15 min treatment using 0.1% TritonX‐100 and overnight incubation with rabbit anti‐Col2α1 antibody (GB12021‐100, Servicebio, China) at 4 °C were carried out. Next, after 1 h incubation with Cy3‐labeled goat anti‐rabbit IgG (GB21303, Servicebio, China) and 5 min staining using DAPI, LSCM (ZEISS, Germany), and ImageJ were used to acquire and analyze the results.

### Induction of Osteoclast Differentiation

Mouse bone marrow‐derived macrophages (BMMs) and RAW264.7 cells were commonly used to induce osteoclast differentiation. Briefly, the tibial and femoral marrow cavities were rinsed, and cells were cultured overnight with double antibody (1%)‐ and FBS (10%)‐contained alpha‐MEM (HyClone).^[^ ^51^ ^]^ The incubation was conducted at 37 °C in a humidified incubator with 5% CO_2_. Adherent cells were removed, and 5 min centrifugation with 2500 rpm was conducted to collect the non‐adherent cells. Collected cells were incubated in a culture medium containing 30 ng mL^−1^ of M‐CSF (R&D Systems) for 48 h to generate pure monocytes/macrophages. Next, replacement of the normal medium with the RANKL (40 ng mL^−1^)‐ and M‐CSF (20 ng mL^−1^)‐contained fresh medium was carried out after two‐day culture. Then, cells were incubated with this medium for 7 days and subjected to anti‐tartrate acid phosphatase (TRAP) staining (Sigma–Aldrich) to assess their activity. Additionally, RANKL was supplemented with AHAMA, CQ‐HA, and SCT‐HA to investigate their impact on osteoclastogenesis. RAW264.7 cells were employed to induce osteoclast differentiation. After 3 days of incubation in the presence of RANKL (40 ng mL^−1^) and M‐CSF (20 ng mL^−1^), total RNA was extracted from RAW264.7 cells and subjected to reverse transcription, followed by RT‐qPCR detection. Primer sequences for PDGF‐BB, NFATc1, Cathepsin K, and TRAP are provided in Table S1 (Supporting Information). The 2^‐ΔΔCT method was applied to process the results, and each experiment was repeated three times for statistical accuracy.

### Mouse OA Model

All animal experiments were approved by the Animal Research Committee of Ruijin Hospital, Shanghai Jiao Tong University (The Ethical Clearance number is SYXK 018–0027). Ten‐week‐old male wild‐type C57BL/6 mice were chosen as the animal model for the experiments. First, mice were anesthetized and prepared for surgery. The anterior cruciate ligament (ACL) was transected by creating an incision in the joint cavity to induce mechanical instability, and then the incision was sutured. The anesthesia and skin surgery without ligament damage comprised the Sham operation. Next, five groups of OA mice were categorized. Equal amounts of PBS, AHAMA, CQ‐HA, and SCT‐HA (100 µL) were injected at days 14 and 35. The group injected with PBS was the control. The diameter of the syringe is 450–500 µm, which could meet the needs of joint cavity injection. The injection dose in the joint cavity was 50 µg mL^−1^ with 100 µL each injection.

### Histochemistry, Immunohistochemistry, and Histomorphometry

Mice were euthanized for 10 weeks postoperatively, and knee samples were collected. Samples were fixed using 4% paraformaldehyde, followed by decalcification, and embedding in paraffin. The sagittal surface of the knee joint was assessed for histopathological features using Safranin O‐fast green, toluidine blue, H&E and TRAP staining. Next, samples were evaluated and scored according to the OARSI criteria. Immunohistochemistry was conducted on paraffin sections using standard protocols. The following antibodies were applied: MMP13 (Abcam, ab39012,1:100) and Col2α1 (Abcam, ab34712 1:100). Sections were then stained with a secondary antibody and DAB substrate.

Tissue sections were immunofluorescent stained and allowed to air dry. Then, 10 min permeabilization using 0.3% Triton X‐100, 30 min blocking using 5% donkey serum at RT, and overnight incubation at 4 °C were carried out. The following primary antibodies include: Endomucin (Santa Cruz Biotechnology, Santa Cruz, sc‐65495,1:100) and CD31 (R&D Systems, Minneapolis, fab3628g,1:100). Briefly, after hybridization with specific primary antibodies, the sections were washed using PBS three times and incubated for 1 h with suitable Alexa Fluor‐conjugated secondary antibody (Molecular Probes, 1:400.) Next, DAPI was used to stain the nuclei.

To label SB mineralization deposits, Calcein green (30 mg kg^−1^, Sigma, USA) and Alizarin red (30 mg kg^−1^, Sigma, USA) were subcutaneously injected after dissolving using sodium bicarbonate (2%). Calcein green was injected 10 days before mice euthanasia, and Alizarin red was injected 2 days before. After removing the muscle and soft tissues surrounding them, Fix the knee joints using 70% alcohol. Next, they were dehydrated using an alcohol gradient (70, 80, 90, and 100%), with each concentration being applied for 2 days (a total of 7 days). After embedding in Polymethylmethacrylate (PMMA), 100 µm thick sections of mouse tibia were obtained using the sagittal cut of the EXAKT cutting and grinding system. These sections were further ground to a thickness of 50 µm. Subsequently, double‐labeled fluorescence with Calcein green and Alizarin red was observed using a fluorescence microscope to access the mineral apposition rate (MAR).

### Gait Acquisition, Micro‐Computed Tomography (CT), and CT‐Based Microangiography Evaluation

Ten weeks post‐operation, the left feet of the mice were submerged in blue ink, while their right feet were immersed in red ink. Subsequently, they were allowed to walk naturally on collection paper to obtain their footprints. Ten weeks postoperatively, knee samples were collected from mice after euthanasia. Arthrography was performed using a high‐resolution micro‐CT imaging system^[^ ^52^ ^]^ (SkyScan1172, BrukerBioSpin, Belgium). Micro‐CT scans and reconstructions were used to assess the following parameters: relative osteophyte volume, SB plate bone mineral density (BMD), trabecular bone pattern factor (Tb. Pf), trabecular bone volume per tissue volume (BV/TV), and SB plate thickness (SBP.Th).

Blood vessels were imaged via angiography on long bones perfused with microfil. Euthanizing each group of mice 10 weeks postoperatively, the thorax was opened, and blood was drained through an incision in the right atrium. A needle injected a saline solution (100 U mL^−1^) containing sodium heparin into the left ventricle, flushing the vascular system. Fixation of the specimens was achieved by perfusing the heart with 10% neutral formalin. After flushing the vessels with a heparinized saline solution to eliminate excess formalin, the vascular system was labeled by injecting an opaque silicone rubber compound containing lead chromate (Microfil MV‐122, Flow Tech, USA). Overnight storage at 4 °C resulted in the polymerization of the contrast agent in the samples. The knee joints of mice were harvested and immersed in 10% neutral buffered formalin for 4 days, ensuring the tissues were fully fixed. Then, the decalcification was performed, and the knee joints were scanned using SkyScan1172. The number of vessels (VN) and the volume (VV/YV) of SB vessels in the knee joint were determined based on micro‐CT scans and reconstructions.

### Transcriptome Sequencing and Data Analysis

Mice were euthanized to collect SB samples from the knee joint after ten weeks postoperatively. In the next step, samples were allocated into three groups: Sham (mice with sham operation), PBS (mice treated with PBS injection), and SCT‐HA (mice treated with SCT‐HA injection). For the PBS and SCT‐HA groups, 100 µL of PBS and SCT‐HA microspheres were injected into the knee joint postoperatively at weeks 2 and 5. Each group had three replicates. The SB samples were rapidly frozen in liquid nitrogen, and total RNA was extracted using Trizol. Libraries were constructed using the NEB Next Ultra II DNA Library Prep Kit (#E7490). RNA‐sequencing (RNA‐seq) analysis was conducted at Shanghai Origingene Bio‐pharm Technology (Shanghai, China).

### Statistical Analysis

All data were repeated three or more times (*n* ≥ 3) and recorded as mean ± standard deviation (SD). Comparisons between two groups were analyzed using the Two‐tailed unpaired t‐test, while comparisons among more than two groups were analyzed using one‐way analysis of variance (ANOVA) followed by Turkey test. Differences in significance were indicated as follows: ^*^ *p* < 0.05, ^**^ *p* < 0.01, ^***^ *p* < 0.001, ^****^ *p* <0.0001. GraphPad Prism 9.0 (GraphPad Software Inc., USA) was used for statistical analysis.

## Conflict of Interest

The authors declare no conflict of interest.

## Author Contributions

G.Z. and P.Z. contributed equally to this work. Z.Z., W.C., and J.X. conceived and supervised the project. G.Z. and P.Z. contributed to the writing of the article and the specific experiment. X.Y. and Q.J. provided support for cell and animal experiments. J.Q. and L.D. corrected grammar in texts and gave advice on writing.

## Supporting information

Supporting Information

## Data Availability

The data that support the findings of this study are available from the corresponding author upon reasonable request.
