# Supplementary material for: Regulating Chondro‐Bone Metabolism for Treatment of Osteoarthritis via High‐Permeability Micro/Nano Hydrogel Microspheres
Source: Adv Sci (Weinh). 2023 Dec 11;11(5):2305023. doi: 10.1002/advs.202305023 (PMC10837371; doi:10.1002/advs.202305023)
Supplement: Supplementary file 1 — Supporting Information [file ADVS-11-2305023-s001.pdf]

ADVANCED SCIENCE
Open Access

Supporting Information

for *Adv. Sci.*, DOI 10.1002/advs.202305023

Regulating Chondro-Bone Metabolism for Treatment of Osteoarthritis via High-Permeability Micro/Nano Hydrogel Microspheres

Guilai Zuo, Pengzhen Zhuang, Xinghai Yang, Qi Jia, Zhengwei Cai, Jin Qi, Lianfu Deng, Zhenhua Zhou, Wenguo Cui* and Jianru Xiao**

Regulating chondro-bone metabolism for treatment of osteoarthritis via high-permeability micro/nano hydrogel microspheres

Guilai Zuo[1,2,3], Pengzhen Zhuang[4,5], Xinghai Yang[2], Qi Jia[2], Zhengwei Cai[4], Jin Qi[4], Lianfu Deng[4], Zhenhua Zhou[2,] ,Wenguo Cui[4,*], Jianru Xiao[1,2,*]*

[1] School of Health Science and Engineering, University of Shanghai for Science and Technology, Shanghai 200093, P. R. China
[2] Department of Orthopaedic Oncology, Changzheng Hospital, Naval Military Medical University, Shanghai 200003, P. R. China
[3] Department of Bone Tumor, The Affiliated Hospital of Qingdao University, No. 59, Haier Road, Qingdao, Shandong 266000, P. R. China
[4] Department of Orthopaedics, Shanghai Key Laboratory for Prevention and Treatment of Bone and Joint Diseases, Shanghai Institute of Traumatology and Orthopaedics, Ruijin Hospital, Shanghai Jiao Tong University School of Medicine, 197 Ruijin 2nd Road, Shanghai 200025, P. R. China
[5]Pharmaceutical Sciences Laboratory, Faculty of Science and Engineering, Åbo Akademi University, Turku 20520, Finland
*Corresponding author. E-Mail: mg63@smmu.edu.cn (Z. Zhou); wgcui80@hotmail.com (W. Cui); jianruxiao83@smmu.edu.cn (J. Xiao).

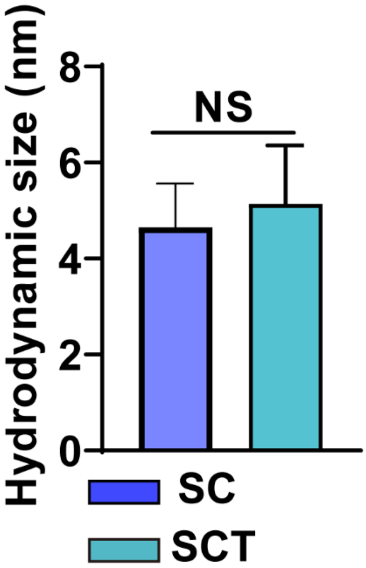

Figure S1. Comparison of diameters between SC and SCT (n = 3). Unpaired Student's t-tests were used to calculate the differences between groups. Results are presented as means ± S.D. A $p < 0.05$ was considered statistically significant; NS: not significant.

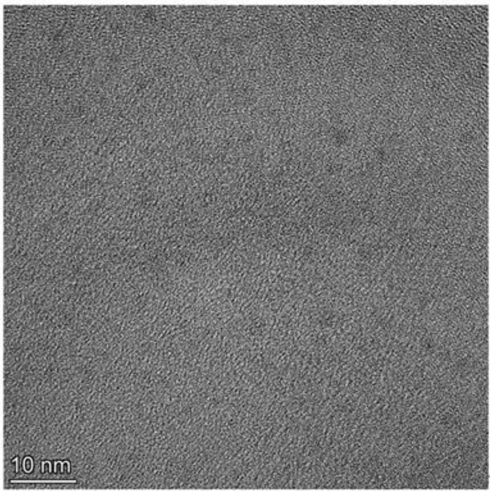

Figure S2. TEM image of the SC.

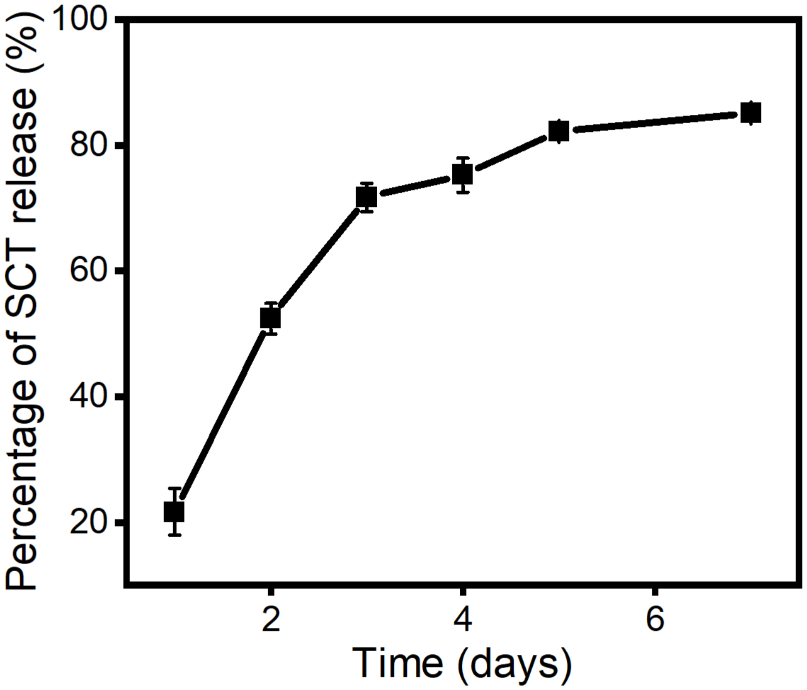

Figure S3. SCT release in a weak acid environment.

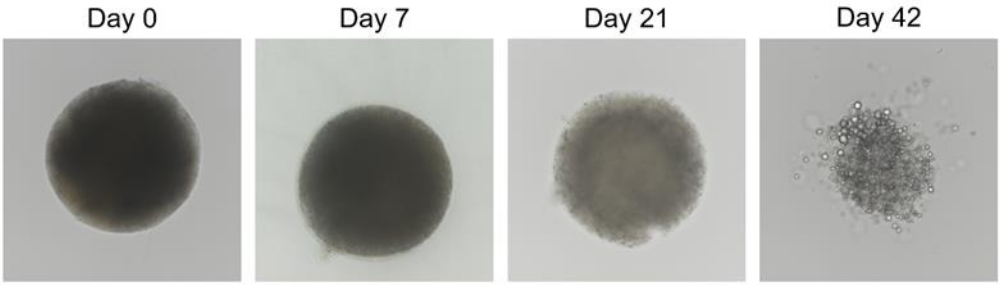

Figure S4. Degradation of SCT hydrogel microspheres.

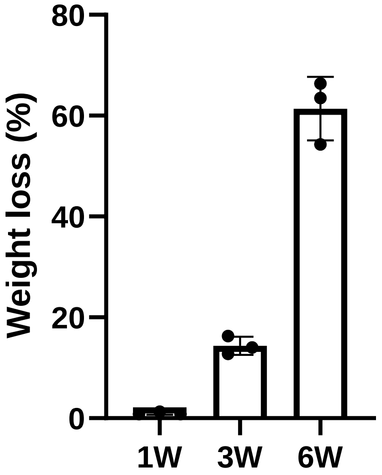

Figure S5. Weight loss of SCT hydrogel microspheres during degradation.

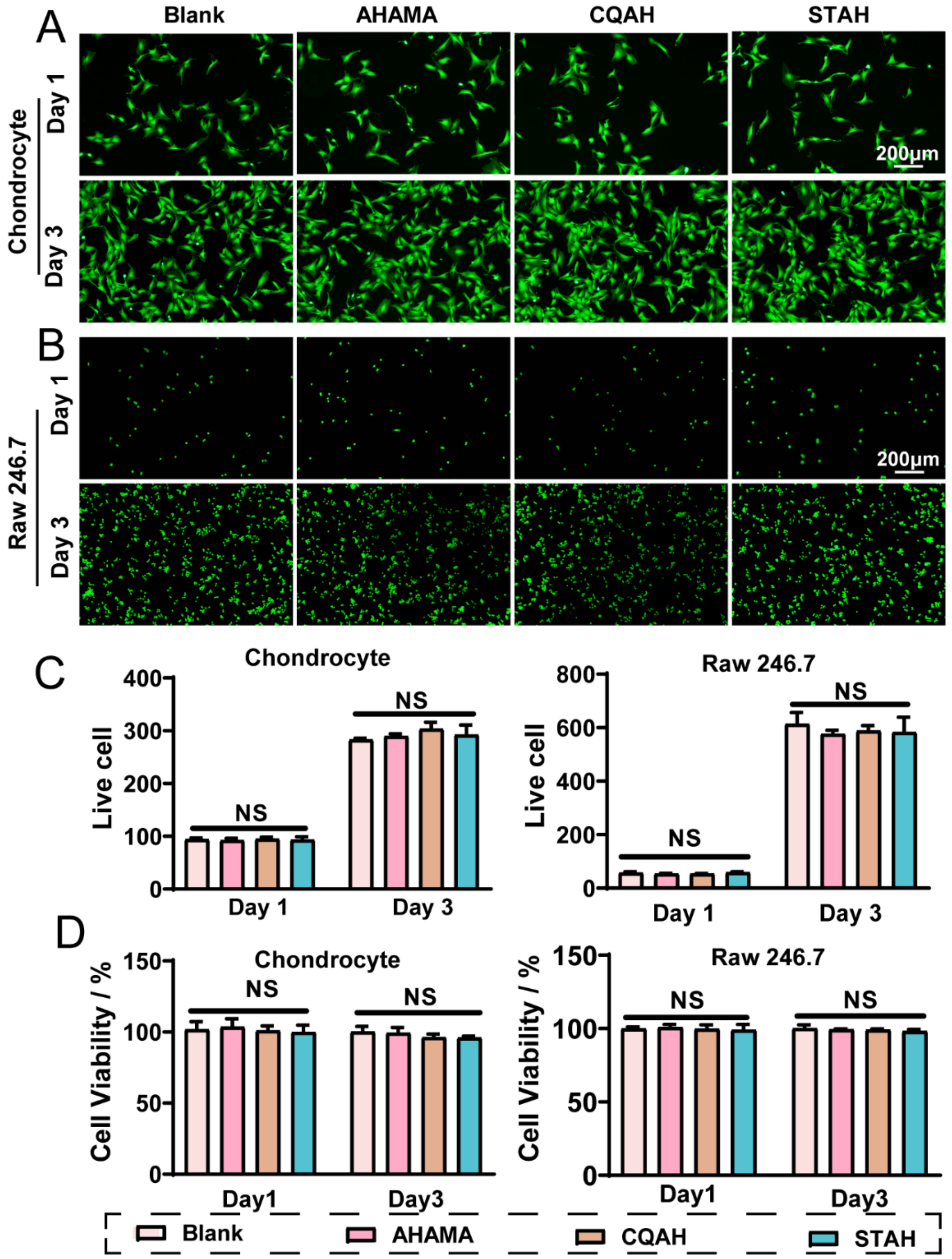

Figure S6. Fluorescence results of A) chondrocytes and B) RAW246.7 cells dead (red)/live (green) staining on days one and three. C) Cell viability counts for chondrocytes and RAW246.7 cells were determined through live/dead staining assays. (n = 3) (NS: not significant). D) CCK-8 results of chondrocytes and RAW246.7 cells on days one and three (n = 3) (NS: not significant) (n = 3 per

group). Data (means ± standard deviations) were analyzed using one-way ANOVA followed by Tukey's posthoc multiple comparison test. NS: not significant.

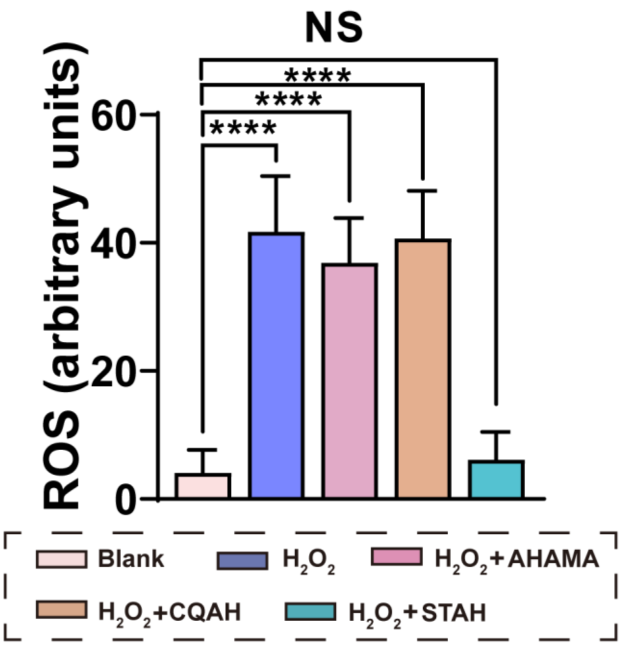

Figure S7: Analysis of DCFH-DA fluorescence intensity in RAW246.7 cells (n = 3 per group). Data (means ± standard deviations) were quantified using one-way ANOVA followed by Tukey's posthoc multiple comparison test. NS: not significant; *$p < 0.05$, **$p < 0.01$, ***$p < 0.001$, and ****$p < 0.0001$.

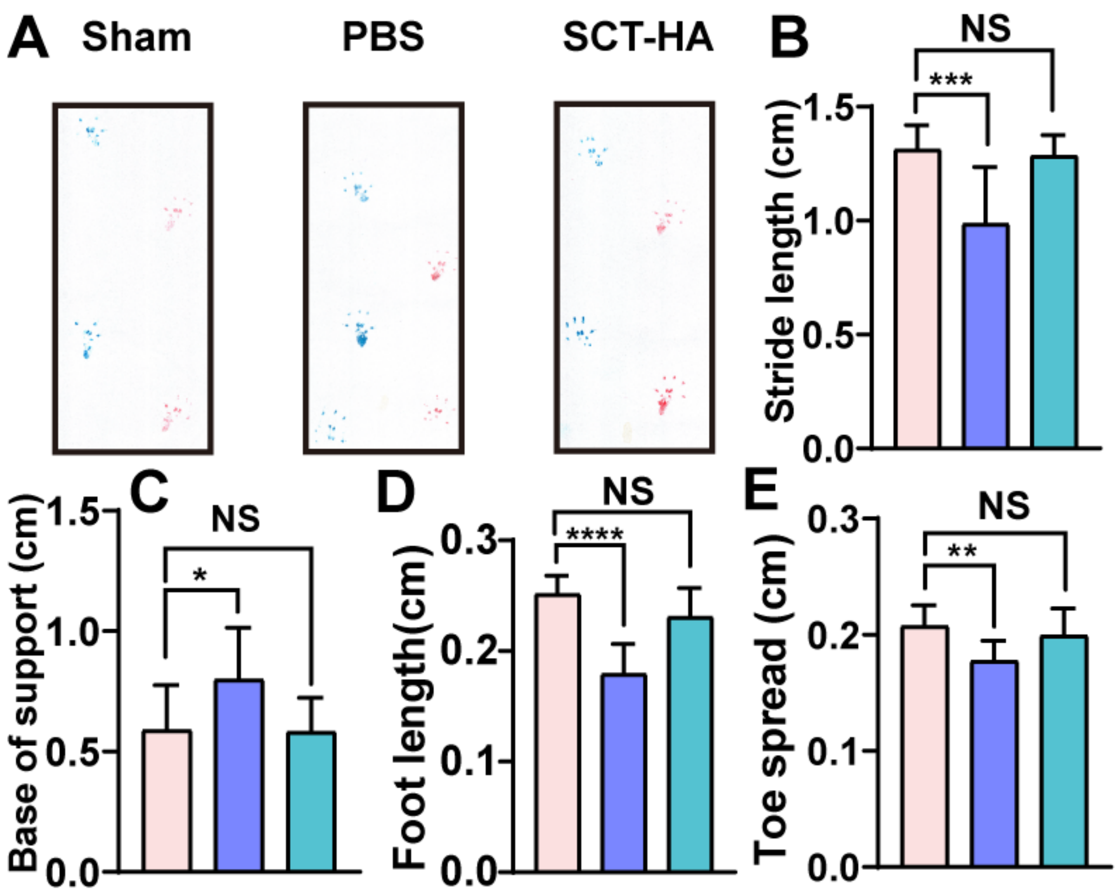

Figure S8. Footprint collection in mice 10 weeks postoperatively. (a) Footprints. Blue: Healthy Side; Red: Modeling Side. (B) Footprint stride length. (C) Footprint base of supports. (D) Foot length. (E) Foot length toe spread (n = 6 per group). Data (means ± standard deviations) were analyzed using one-way ANOVA followed by Tukey's posthoc multiple comparison test. NS: not significant. *p < 0.05, **p < 0.01, ***p < 0.001, and ****p < 0.0001.

Table S1 Primer sequences for real-time PCR studies

GAPDH	FORWARD	AGGTCGGTGTGAACGGATTTG
	REVERSE	TGTAGACCATGTAGTTGAGGTCA
COL2	FORWARD	ATGAGGGAGCGGTAGAGACC
	REVERSE	GCCCTAATTTTCGGGCATCC
Aggrecan	FORWARD	CATCACAGAGTCCGAGTGGG
	REVERSE	ATTGCTCCTGGTCTGCAACG
MMP-13	FORWARD	CTTCTTCTTGTTGAGCTGGACTC

	REVERSE	CTGTGGAGGTCACTGTAGACT
IL-6	FORWARD	TAGTCCTTCCTACCCCAATTTCC
	REVERSE	TTGGTCCTTAGCCACTCCTTC
ADAMTS-5	FORWARD	GGAGCGAGGCCATTTACAAC
	REVERSE	CGTAGACAAGGTAGCCCACTTT
TRAP	FORWARD	CGGTATCAGTGGTCTCAGTGGC
	REVERSE	GCCCTAATTTTCGGGCATCC
Nfatc1	FORWARD	TGGGAGATGGAAGCAAAGACTG
	REVERSE	CAGACATAGAAACTGACTTGGACGG
Cathepsin K	FORWARD	TTACTCCAGTCAAGAACCAGGGC
	REVERSE	GCCTCCACAGCCATAATTCTCA
Pdgf-BB	FORWARD	ACCACTCCATCCGCTCCTTT
	REVERSE	TCGGGTCATGTTCAAGTCCA
